# New quinoline-based triazole hybrid analogs as effective inhibitors of α-amylase and α-glucosidase: Preparation, *in vitro* evaluation, and molecular docking along with *in silico* studies

**DOI:** 10.3389/fchem.2022.995820

**Published:** 2022-09-15

**Authors:** Yousaf Khan, Shahid Iqbal, Mazloom Shah, Aneela Maalik, Rafaqat Hussain, Shoaib Khan, Imran Khan, Rami Adel Pashameah, Eman Alzahrani, Abd-ElAziem Farouk, Mohammed Issa Alahmdi, Hisham S. M. Abd-Rabboh

**Affiliations:** ^1^ Department of Chemistry, COMSATS Universityislamabad Campus, Islamabad, Pakistan; ^2^ Department of Chemistry, School of Natural Sciences (SNS), National University of Science and Technology (NUST), Islamabad, Pakistan; ^3^ Department of Chemistry, Abbottabad University of Science and Technology (AUST), Abbottabad, Pakistan; ^4^ Department of Chemistry, Hazara University, Mansehra, Pakistan; ^5^ Department of Chemistry, Faculty of Applied Science, Umm Al-Qura University, Makkah, Saudi Arabia; ^6^ Department of Chemistry, College of Science, Taif University, Taif, Saudi Arabia; ^7^ Department of Biotechnology College of Science, Taif University, Taif, Saudi Arabia; ^8^ Department of Chemistry, Faculty of Science, University of Tabuk, Tabuk, Saudi Arabia; ^9^ Chemistry Department, Faculty of Science, King Khalid University, Abha, Saudi Arabia; ^10^ Department of Chemistry, Faculty of Science, Ain Shams University, Cairo, Egypt

**Keywords:** quinoline, triazole, α-amylase enzymes, α-glucosidase enzymes, molecular docking

## Abstract

The 7-quinolinyl-bearing triazole analogs were synthesized **(1d–19d)** and further assessed *in vitro* for their inhibitory profile against α-amylase andα-glucosidase. The entire analogs showed a diverse range of activities having IC_50_ values between 0.80 ± 0.05 µM to 40.20 ± 0.70 µM (α-amylase) and 1.20 ± 0.10 µM to 43.30 ± 0.80 µM (α-glucosidase) under the positive control of acarbose (IC_50_ = 10.30 ± 0.20 µM) (IC_50_ = 9.80 ± 0.20 µM**)** as the standard drug. Among the synthesized scaffolds, seven scaffolds **12d**, **10d**, **8d**, **9d**, **11d**, **5d**, and **14d** showed excellent α-amylase and α-glucosidase inhibitory potentials with IC_50_ values of 4.30 ± 0.10, 2.10 ± 0.10, 1.80 ± 0.10, 1.50 ± 0.10, 0.80 ± 0.05, 5.30 ± 0.20, and 6.40 ± 0.30 µM (against α-amylase) and 3.30 ± 0.10, 2.40 ± 0.10, 1.20 ± 0.10, 1.90 ± 0.10, 8.80 ± 0.20, 7.30 ± 0.40, and 5.50 ± 0.10 µM (against α-glucosidase), respectively, while the remaining 12 scaffolds **19d**, **8d**, **17d**, **16d**, **15d**, **7d**, **4d**, **3d**, **1d**, **2d**, **13d** and **6** **d** showed less α-amylase and α-glucosidase inhibitory potentials than standard acarbose but still found to be active. Structure–activity connection studies also showed that scaffolds with electron-withdrawing groups like -Cl, -NO_2_, and -F linked to the phenyl ring had higher inhibitory potentials for -amylase and -glucosidase than scaffolds with -OCH_3_, -Br, and -CH_3_ moieties. In order to better understand their binding sites, the powerful scaffolds **11d** and **9d** were also subjected to molecular docking studies. The results showed that these powerful analogs provide a number of important interactions with the active sites of both of these targeted enzymes, including conventional hydrogen bonding, pi–pi stacking, pi–sulfur, pi–anion, pi–pi, pi–sigma, T-shaped, and halogen (fluorine). Furthermore, various techniques (spectroscopic), including 1H, ^13^C-NMR, and HREI-MS mass, were used to explore the correct structure of newly afforded hybrid scaffolds based on quinoline-bearing triazole ring.

## 1 Introduction

In the past few decades, DM (diabetes mellitus) was known to be the most familiar epidemic disorder, which adversely affects the metabolic functions performed by the endocrine system. It is growing and spreading rapidly all over the world. Islets of Langerhans are well-known specialized cells that are responsible for producing insulin in the human body when a diabetic patient does not either produce insulin or is incapable of consuming insulin properly. Due to this dysfunction, the glucose (sugar) level of blood increases in the body, resulting in hyperglycemia. DM is categorized into two types, insulin-reliant, which is due to a lack of production of insulin in the human body termed type-1; however, in type-2 diabetes, either insulin is not produced by the human body or is incapable of utilizing insulin properly. After cardiovascular and cancer diseases, which result in mortality, DM is among the most frequent and rapidly growing chronic diseases (Li et al., 2004; Chauhan et al., 2010). However, inhibition of α-amylase and α-glucosidase, as being carbolytic enzymes, is one of the common approaches to minimizing or managing these metabolic disorders (Zaharudin et al., 2019). Lipase, protease, and amylase are the most used enzymes in food biotechnology and industry (Asgher et al., 2007). α-amylase, which belongs to the class of metalloenzymes, catalyzes the formation of glucose and maltose through hydrolysis of starch (polysaccharides) (Sundarram and Murthy, 2014; Taha et al., 2017). The human body absorbs starch through the sequential catalytic action of α-glucosidase (intestinal) andα-amylase (Asgher et al., 2007). Inhibitors of α-amylase and α-glucosidase play a crucial role in modulating the glucose level of blood after taking a meal. Therefore, the α-amylase and α-glucosidase inhibitory potential can be clinically utilized as chemotherapeutic agents and finds applications in the treatment of DM, in addition to obesity (Gunawan-Puteri et al., 2012; Taha et al., 2017).

In addition to the usage of several approaches, the inhibitors of α-glucosidase are used as a therapeutic approach for the treatment of DM (Jong-Anurakkun et al., 2007). The α-glucosidase inhibitors slow down the absorption of glucose, therefore resulting in a decrease in the digestion process. Miglitol and acarbose are important inhibitors of the α-glucosidase enzyme commonly used to decrease the glucose level of postprandial blood. These synthesized inhibitors inhibit glucose formation in the small intestine through inhibition of the α-glucosidase enzyme. Acarbose, an antidiabetic drug with α-amylase and α-glucosidase inhibitory potentials, is currently used in type-2 DM treatment (Salar et al., 2017). Nonetheless, different side effects, such as abdominal pain and flatulence, are associated with acarbose. The use of only drugs is often limited by these side effects; consequently, the efficiency was improved by using in combination with other antidiabetic drugs. Therefore, designing and constructing more active scaffolds having fewer side effects to treat DM is time-consuming (Meneilly et al., 2000).

Quinoline scaffolds find application in numerous pharmaceutically potent drugs and received much attention in the field of medicinal chemistry. Quinoline scaffolds were reported to show a broad range of biological activities such as antifungal (Tabassum et al., 2021), antibacterial (Ghosh et al., 2020), antileishmanial (Gupta et al., 2021), anticonvulsant (Al-Ostoot et al., 2021), anti-Zaka virus (Taha et al., 2019), antimalarial (Vinindwa et al., 2021), proton pump inhibitors (Cernicchi et al., 2021), antiproliferative (Ammar et al., 2021), antiulcer (Atukuri et al., 2020), antidiabetic (Taha et al., 2019), antimicrobial, anti-inflammatory (Douadi et al., 2020), antitrypanosomal (Zhang et al., 2018), antitubercular (Nesaragi et al., 2021), antioxidant agent (Velmurugan et al., 2021), and antihypertensive (Kumari et al., 2019).

It is well known that scaffolds harboring the 1,2,4-triazole motif have a variety of biological and pharmacological properties, including anticonvulsant, anti-inflammatory, antiproliferative, and antifungal activities (Fadaly et al., 2020; Jahani et al., 2020; Joshi et al., 2020). Furthermore, 1,2,4-triazole is a pharmacologically active scaffold that is crucial for therapeutic candidates such as anastrozole, vorozole, and letrozole, all of which have been shown to be efficient aromatase inhibitors and have applications in the treatment of breast cancer (Ammazzalorso et al., 2021). Additionally, it was shown that 1,2,4-triazole containing mercapto scaffolds have antitubercular, antibacterial, antifungal, and anticancer effects (Mallikarjuna et al., 2009; Pitucha et al., 2020).

**FIGURE 1:**
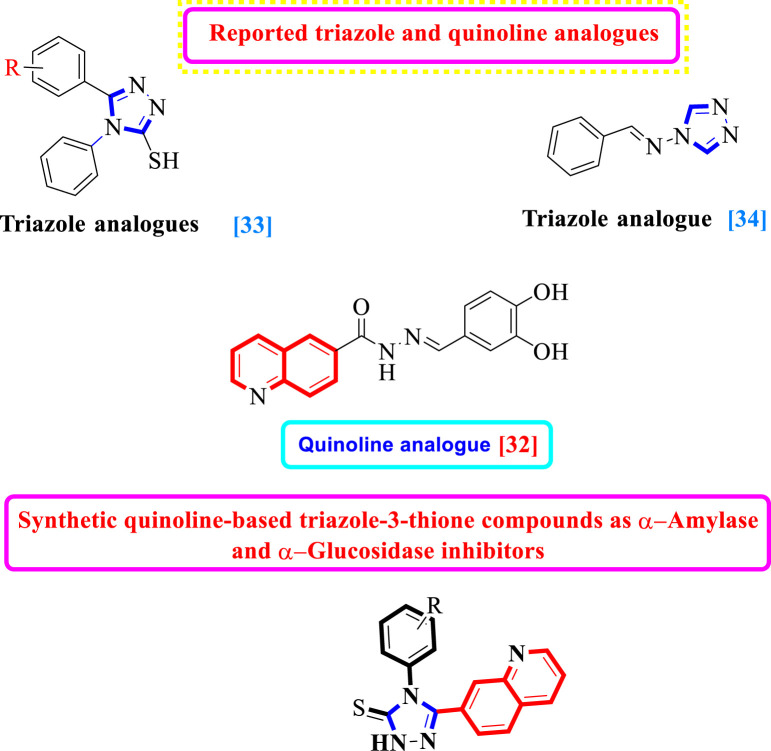
Hybridization of the quinoline moiety with a triazole ring.

In the recent past, researchers had investigated numerous heterocyclic moieties of different categories and classes to explore their inhibitory profile in search of lead molecules. Keeping in view the α-amylase and α-glucosidase profile of quinoline and triazole-3-thione analogs, herein in this study, we have incorporated two heterocyclic rings in the same molecule as7-quinolinyl-bearing triazole-3-thione analogs **(1d–19d)** with the hope of further expanding their inhibitory potentials as dual α-amylase and α-glucosidase inhibitors (Figure 1).

## 2 Results and discussion

### 2.1 Chemistry

**SCHEME 1:**
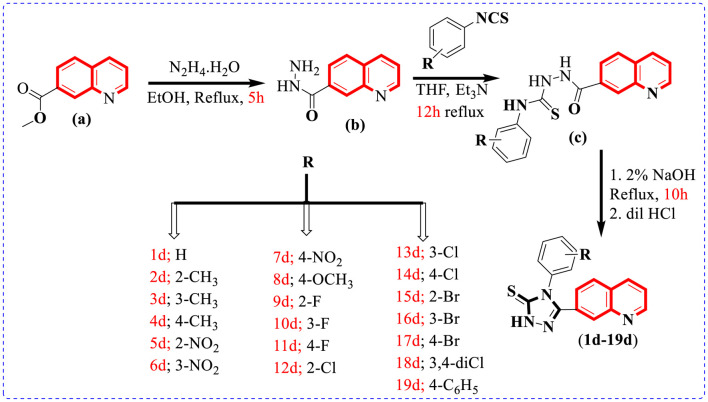
Preparation of 7-quinolinyl-based triazole-3-thione derivatives (1d–19d).

To afford new hybrid scaffolds **(1d**–**19d)** based on the quinoline-bearing triazole skeleton, methyl quinoline-7-carboxylate (0.5 mmol) (a) solution stirred in EtOH (10 ml) was reacted and refluxed with hydrazine hydrate (1 equivalent) for 5 h. As the reaction was completed, a solid residue (0.5 mmol) was obtained by evaporation of the solvent and then washed with cold water and dried to afford quinoline-7-carbohydrazide (b). In the second step, the mixture of quinoline-7-carbohydrazide (0.5 mmol), the corresponding phenyl isothiocyanates (1 equivalent), and Et_3_N (1 ml) in THF (10 ml) was refluxed for 12 h and then quenched with water (10 ml) to form a precipitate, which was further filtered and using EtOH (5 ml) recrystallized to access an intermediate, which further underwent cyclization with 2% NaOH (10 ml) followed by neutralization with dilute HCl (5 ml) to afford the targeted quinoline-based triazole **(1d**–**19d)** scaffolds in the appropriate yield (Scheme 1) Table 1.

**TABLE 1:** Inhibition profile (*in vitro*) of targeted α-amylase and α-glucosidase enzymes of hybrid scaffolds of quinoline-based triazole (1d–19d).

Synthetic compound	Substituent	IC_50_ ± SEM^a^ [*μ*M]( α-amylase)	IC_50_ ± SEM^a^ [*μ*M]( α-glucosidase)
**1d**	-H	40.20 ± 0.70	43.30±0.80
**2d**	2-CH_3_	22.60 ± 0.60	25.50 ± 0.60
**3d**	3-CH_3_	32.30 ± 0.60	31.60 ± 0.70
**4d**	4-CH_3_	21.60 ± 0.40	22.50±0.40
**5d**	2-NO_2_	5.30 ± 0.20	8.80 ± 0.20
**6d**	3-NO_2_	21.40 ± 0.30	19.50 ± 0.40
**7d**	4-NO_2_	11.60 ± 0.20	14.60 ± 0.30
**8d**	4-OCH_3_	29.40 ± 0.60	32.10 ± 0.60
**9d**	2-F	1.50 ± 0.10	1.90 ± 0.10
**10d**	3-F	2.10 ± 0.10	2.40 ± 0.10
**11d**	4-F	0.80 ± 0.05	1.20 ± 0.10
**12d**	2-Cl	4.30 ± 0.10	5.50 ± 0.10
**13d**	3-Cl	14.60 ± 0.30	14.60 ± 0.40
**14d**	4-Cl	6.40 ± 0.30	7.30 ± 0.40
**15d**	2-Br	17.20 ± 0.30	19.10 ± 0.20
**16d**	3-Br	31.80 ± 0.60	33.70 ± 0.60
**17d**	4-Br	19.20 ± 0.30	23.50 ± 0.40
**18d**	2,4-diCl	1.80 ± 0.10	3.30 ± 0.10
**19d**	4-C_6_H_5_	18.50 ± 0.30	19.30±0.40
Standard acarbose drug	10.30 ± 0.20	9.80 ± 0.20	

### 2.2 Biological activity

#### 2.2.1 Inhibition study of α-amylase and α-glucosidase

In this study, nineteen scaffolds based on quinoline-bearing triazole were afforded and then assessed against α-amylase and α-glucosidase to explore their inhibition profile. The entire analogs showed a diverse range of activities having IC_50_ values between 0.80 ± 0.05 µM and 40.20 ± 0.70 µM (α-amylase) and 1.20 ± 0.10 µM and 43.30 ± 0.80 µM (α-glucosidase) under the positive control of acarbose [(IC_50_ = 10.30 ± 0.20 µM) (IC_50_ = 9.80 ± 0.20 µM)] as the standard drug. Among the synthesized scaffolds, seven scaffolds **12d**, **10d**, **18d**, **9d**, **11d**, **5d** and **14d** showed superior α-amylase and α-glucosidase inhibitory potentials than standard acarbose drugs with IC_50_ values of 4.30 ± 0.10, 2.10 ± 0.10, 1.80 ± 0.10, 1.50 ± 0.10, 0.80 ± 0.05, 5.30 ± 0.20, and 6.40 ± 0.30 µM (against α-amylase) and 3.30 ± 0.10, 2.40 ± 0.10, 1.20 ± 0.10, 1.90 ± 0.10, 8.80 ± 0.20, 7.30 ± 0.40, and 5.50 ± 0.10 µM (against α-glucosidase), respectively, while the remaining 12 scaffolds **19d**, **8d**, **17d**, **16d**, **15d**, **7d**, **4d**, **3d**, **1d**, **2d**, **13d** and **6d** showed less α-amylase and α-glucosidase inhibitory potentials than standard acarbose but still found to be active. SAR studies suggest that the entire part of synthetic scaffolds, including quinoline, triazole ring, and *N*-phenyl ring, actively participates in the activity, and any change observed in the activity was due to alteration in position, number/s, and nature of substituents around phenyl ring **B**.

##### 2.2.1.1 Structure–activity relationship study for α-amylase enzyme

It was noteworthy from SAR studies that quinoline-based triazole scaffolds that hold the fluoro moiety as being the electron-withdrawing entity attached to various positions of the aryl ring show a significant role in the inhibition of the α-amylase enzyme, and hence hybrid scaffolds based on the quinoline-bearing triazole moiety showed better inhibition than -CH_3_, -OCH_3,_ and–Br groups bearing scaffolds. This enhanced inhibitory potential of fluoro moiety-bearing analogs was due to greater electron withdrawing affinity of the –F moiety, which makes the ph-ring more effective for interactions with α-amylase active pocket. Moreover, it also seemed that the activity was altered by bringing variation in the position of the –F moiety around aryl ring **B**. In current newly afforded scaffolds, the analog **11d** that holds the –F moiety at 4-position of aryl ring **B** linked to the *N*-triazole skeleton was found to show ten-fold enhanced potency than standard acarbose drug. However, analogs **9d** with *ortho*-F and **10d** having *meta*-F substitutions displayed less potency than scaffold **11d**, although these analogs resemble in their structure with analog **9d** but have different positions around phenyl ring **B**. This variation in potency of these–F moiety-bearing scaffolds was due to different positions of the -F moiety at the phenyl ring, indicating that alteration in the position of the –F moiety may result in diverse potency.

**FIGURE 2:**
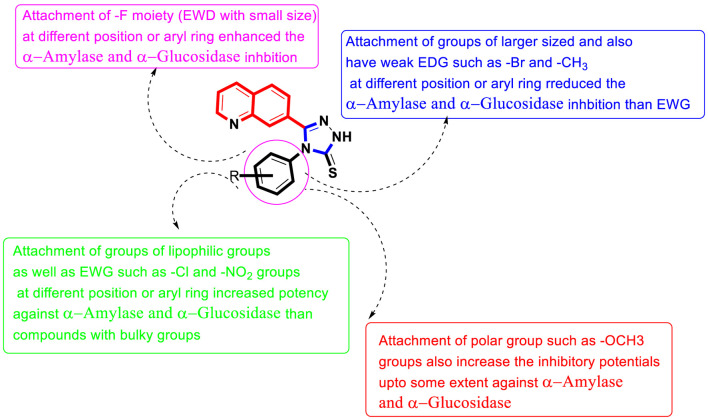
Summary of SAR studies of quinoline-based triazole against α-amylase and α-glucosidase enzymes.

Similarly, the alteration in the position of the attached substitute also affects other–Cl moiety-bearing scaffolds **14d**, **12d**, and **13d**. By comparing scaffold **12d** that holds *ortho*–Cl moiety with scaffolds **14d** bearing *para*-Cl and **13d** having *meta*-Cl substitutions, the *ortho*-Cl moiety holding analog **12d** showed enhanced potency than its counterparts **14d** and **13d**, although these scaffolds are structurally similar. This indicates that the activity was diminished by shifting *ortho*-Cl moiety either to *para*- or *meta*-position as in scaffolds **14d** and **13d**. In addition, compound **14d** bearing *para*-Cl substitution showed 2-folds more potency than scaffold **7d** bearing *para*-NO_2_ moieties on the aryl ring, although the position of substituents around the aryl ring is the same. The activity was different in the case of both these compounds, which may be owing to the diverse nature of attached substituents (-Cl and–NO_2_). Moreover, by comparing the *ortho-*Cl moiety bearing scaffold **12d** with compound **5d** that hold the *ortho*-NO_2_ group on the aryl ring of the triazole skeleton, the scaffold **12d** has emerged as a better inhibitor of α-amylase than scaffold **5d**. This difference in potency between these analogs was caused by the unique properties of both the -Cl and -NO_2_ moieties. If we compared compound **14d** (IC_50_ = 6.40 ± 0.30 µM) bearing mono-chloro substitution on *4*-position of the attached *N*-ph-ring with its counterpart 2 bearing di-Cl substitution linked to *3*-and *4*-position of the phenyl ring, the compound **18d** showed better activity than compound **14d** due to the attached di-Cl substitutions that offered stronger electron-withdrawing effect. Analog **1d** having un-substituted attached ph-ring is recognized as the least potent inhibitor among the newly constructed scaffolds. However, the activity may be enhanced when substituents of either electron-withdrawing/electron-donating properties are attached to it at various positions of the ph-ring. Therefore, compounds **8d** (having *para*–OCH_3_), **4d** (having *para*–CH_3_), **14d** (having (having *para*–Cl), and **18d** (having *3,4*–di-Cl) moieties showed enhanced α–amylase inhibitory potentials when compared to compound **1d** with un-substituted ph-ring, indicating that the activity was largely dependent on attached electron-withdrawing/electron-donating groups (Figure 2).

##### 2.2.1.2 Structure–activity relationship study for α-glucosidase enzyme

Among halogen-substituted analogs, fluoro-substituted analogs (**10d, 11d** and **9d**) showed enhanced α–glucosidase inhibitory potentials when compared to Cl-substituted (**14d**, **12d** and **13d**) and bromo-substituted analogs (**17d**, **16d** and **15d**). Similarly, Cl-substituted scaffolds display better activity than bromo-substituted analogs. This shows that the α–glucosidase activity was enhanced by increasing the electron-withdrawing nature of the attached substituent; therefore, halogen-substituted scaffolds showed activity in order of–F > Cl > Br. Moreover, by comparing the *para*-F moiety bearing analog **11d** with compounds **10d** bearing *meta*-F and **9d** having *ortho*-F group, compound **11d** showed superior potentials than its counterparts 10d and **9d**, which are similar in structure to compound **11d**. This difference in activity found in these fluoro-substituted analogs was due to the different positions of the attached electron-withdrawing fluoro moiety around the ph-ring. If we compare analog **14d** and **12d** with mono-chloro substitution with compound **18d** bearing 3, 4-di Cl chloro groups, the di-Cl moieties bearing scaffold 18d showed superior activity than a mono-Cl moiety containing scaffolds **14d** and **12d**. This enhanced activity of compound **18d** may be due to the stronger electron-withdrawing effect offered by the two chloro groups as compared to compound **14d** and **12d** bearing mono-chloro substitution. Similarly, by comparing scaffold **5d** bearing *ortho*-nitro substitution with its structurally similar analog **7d** that holds the nitro group at the *para*-position of the phenyl ring, the compound **5d** showed enhanced activity than its counterpart **7d**. This shows that alteration in the position of substitution around the phenyl ring greatly affects the enzymatic activity (Figure 2).

Based on the aforementioned observation, it was concluded that the inhibitory potentials of both α-amylase and α-glucosidase were largely dependent on substitution patterns around the ph-ring attached to the hybrid triazole-based quinoline skeleton. Moreover, it was also noted that the activities changed by bringing alterations in nature, position, and number/s of attached substituents.

### 2.2 Experimental

The experimental data (general procedure, spectral analysis, α-amylase, and α-glucosidase inhibition, and docking protocols (Li et al., 2015; Mrabti, 2018; Rao et al., 2021) were included in the supporting documentation.

## 3 Molecular docking

To explore the active part in newly constructed quinoline-based triazole scaffolds against α-amylase and α-glucosidase, molecular docking was carried out. Among newly afforded scaffolds being tested, the analogs that hold fluoro moiety showed enhanced inhibitory potentials (*in vitro*), and hence these fluoro-substituted analogs were subjected to the molecular docking study to explore their binding site and results obtained from molecular docking show that the most potent analogs **11d** and **9d** not only display better potency (*in vitro*) but also furnish numerous important interactions (*in silico*) with the active site of the amino acid.

**FIGURE 3:**
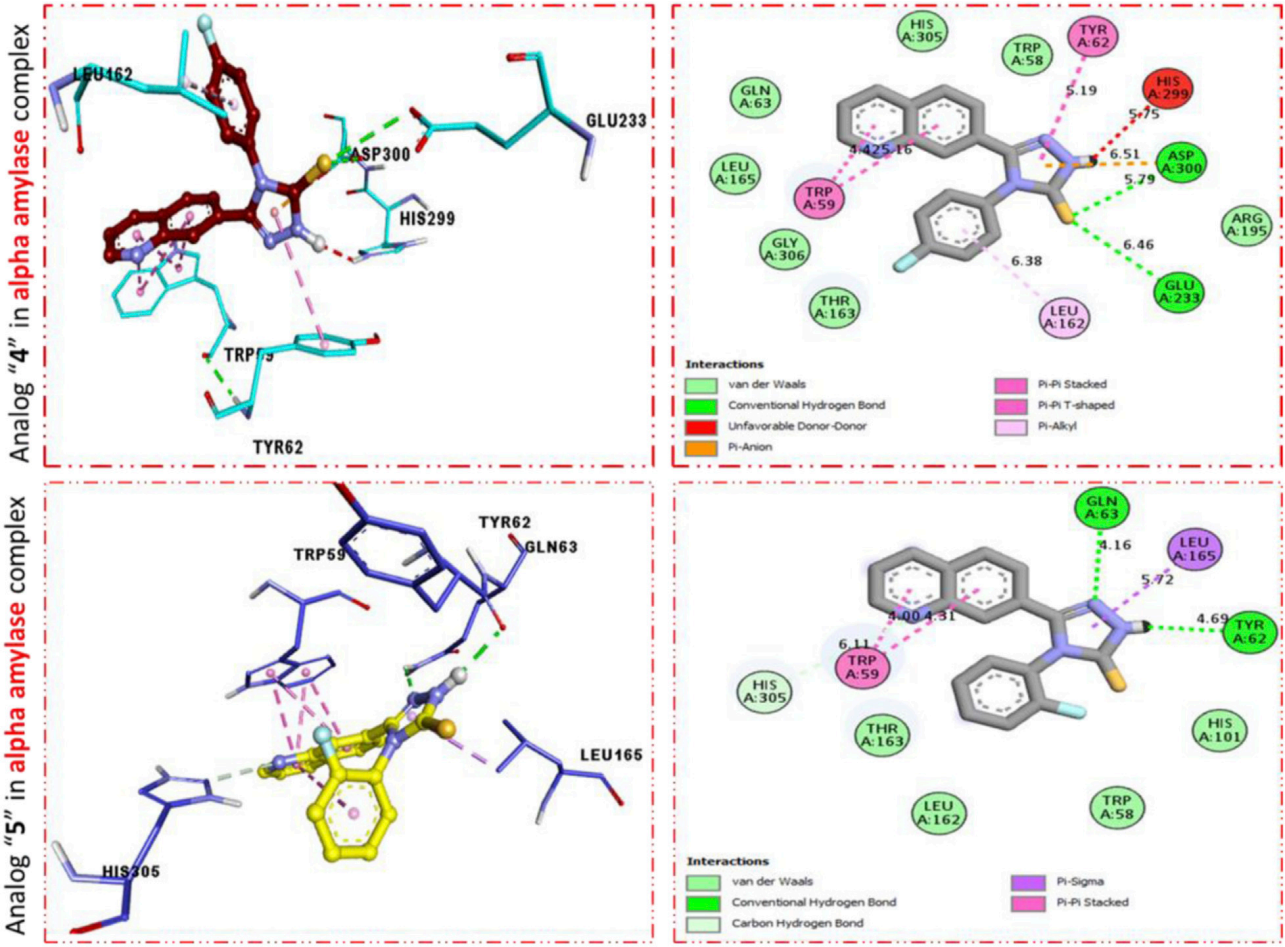
PLI profile indicates the corresponding surface of α-amylase enzyme and its interactions with fluoro-substituted scaffolds 11 d and 9d.

It was shown by the PLI profile of potent analog **11d** bearing–F moiety that analog 11d against α-amylase displays various important interactions (Figure 3) such as TYR-62 (π–π stacked), HIS-299 (donor interactions), ASP-3002 (H-B), GLU-233 (H-B), LEU-162 (π–R), HIS-305 (donor interactions), and TRP-59 (π–π T-shaped). Similarly, analog **9d** in α-amylase showed various interactive residues from a certain distance, while the residues are TRP-59 (π–π stacked), GLN-63 (H-B), LEU-165 (π–sigma), and TRY-62 (H-B) as shown in Figure 3.

**FIGURE 4:**
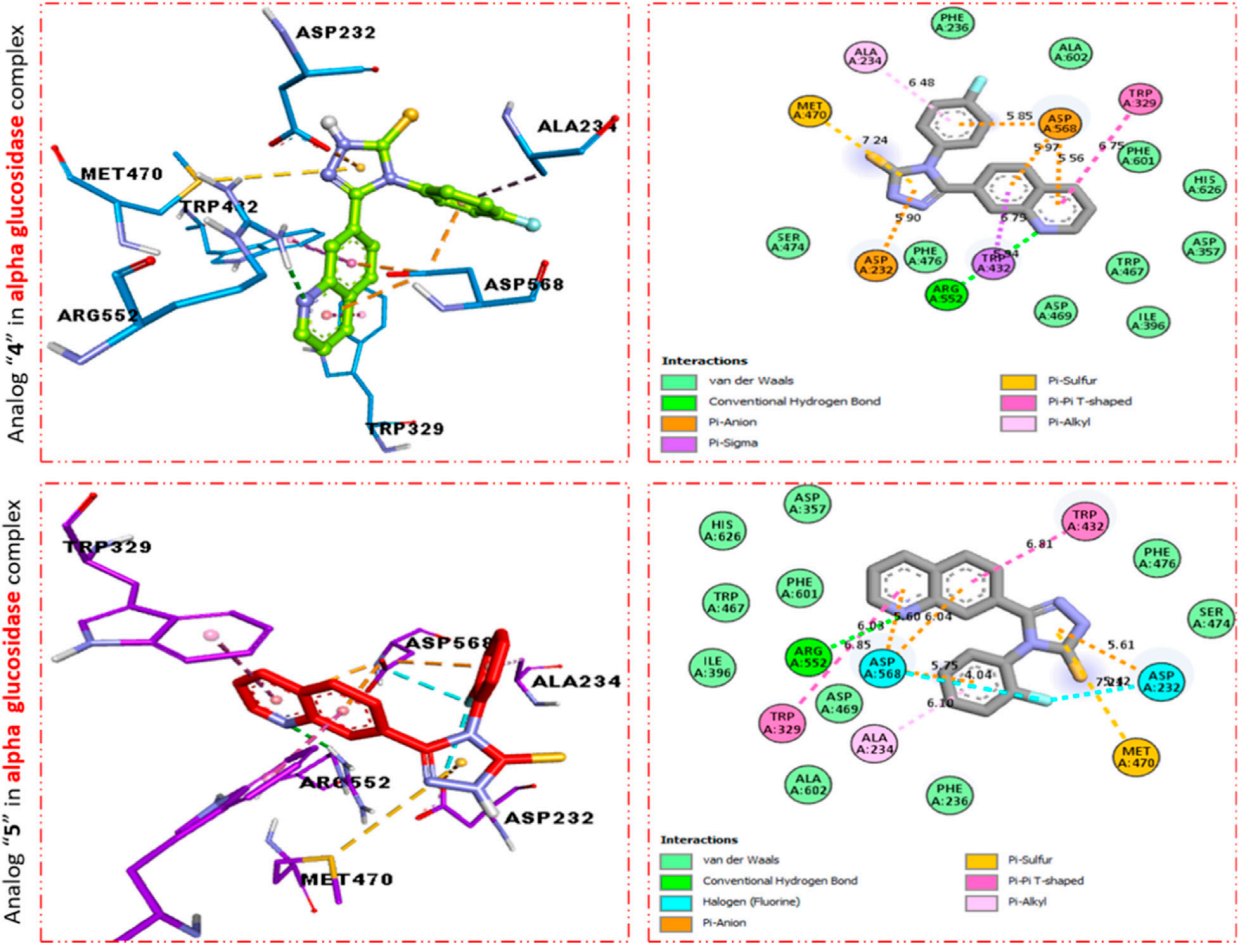
PLI profile indicates the corresponding surface of α-glucosidase enzyme and its interactions with fluoro-substituted scaffolds 11 d and 9d.

Furthermore, scaffold 4 also displays better interactions against the α–glucosidase enzyme. This analog **11d** having *para*-F moiety on the extended aryl ring interacts with α-glucosidase *via* several interactions, including MET-470 (π–S), ALA-234 (π–R), ASP-568 (π–anion), TRP-329 (π–π T-shaped), TRP-432 (π-sigma), ARG-552 (H-B), and ASP-232 (π–anion) as shown in Figure 4, while analog **9d** exhibited the following interactions against alpha glucosidase such as TRP-432 (π–π T-shaped), ASP-232 (H-F), MET-470 (π–S), ALA-234 (π–R), ASP-568 (H-F), TRP-329 (π–π T-Stacked), and ARG-552 (H-B) along with van der Waals interaction (Figure 4).

It was observed by comparison of the protein–ligand interaction profile of most active scaffolds **11d** and **9d** with protein–ligand interaction of standard acarbose drugs against α-amylase and α-glucosidase enzymes that the active scaffolds **11d** and **9d** established some additional interactions such as halogen (fluorine), π–π T-shaped, π–sulfur, π–alkyl, π–sigma, π–π stacking, and π–anion interactions with the active part of the amino acid of α-amylase and α-glucosidase than standard acarbose drug which furnish only conventional HB van der Waals and carbon HB interactions. Therefore, scaffolds **11d** and **9d** were found to be more potent against α-amylase and α-glucosidase than acarbose standard drugs.

**FIGURE 5:**
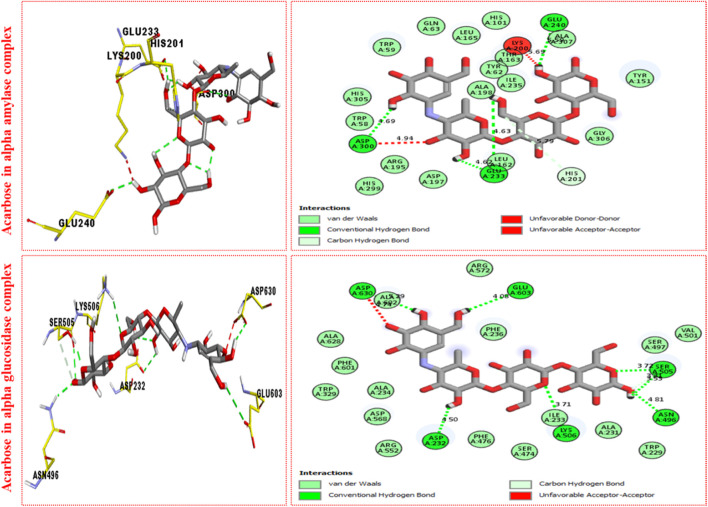
PLI profile indicates the corresponding surface of α-amylase and α-glucosidase enzymes and their interactions with the standard acarbose drug.

The most potent scaffolds at **11d** and **9d** have different potency (*in vitro*) for both enzymes being targeted and hence established a diverse range of interactions (molecular docking) with active sites of these enzymes (Tables 2–4). This difference in activity and different interactions (*in silico*) possessed by these scaffolds toward enzymes being targeted was due to different positions of the attached fluoro moiety around the aryl part of the quinoline-triazole skeleton. It was shown (Figure 5) from docking results that these potent analogs **11d** and **9d** (possess the–F at a different position) furnish a better binding mode of interactions with the active part of enzymes being targeted (α-amylase and α-glucosidase) than standard acarbose drug which established only conventional HB, van der Waals, and carbon HB interactions with the active part of targeted enzymes (α-amylase and α-glucosidase).

**TABLE 2:** RMSD values of different poses of analog 11d against α-amylase and α-glucosidase enzymes.

Mode	Affinity	Distance from best mode	
	(kcal/mol)	rmsd I.b	rmsd u.b
1	−8.2	0.000	0.000
2	−7.9	2.246	3.655
3	−7.9	3.364	6.471
4	−7.8	2.030	2.595
5	−7.6	3.308	5.323
6	−7.2	3.685	6.603
7	−7.2	3.019	4.418
8	−7.2	4.254	7.764
9	−7.0	2.865	3.903
Analog-5 RMSD values of different poses against alpha-amylase			

Mode	Affinity	Distance from best mode	
	(kcal/mol)	rmsd I.b	rmsd u.b
1	−8.5	0.000	0.000
2	−7.7	2.667	3.468
3	−7.3	8.027	10.161
4	−7.0	3.214	5.774
5	−7.0	2.981	5.877
6	−6.8	2.022	2.520
7	−6.8	4.092	5.322
8	−6.8	3.757	6.614
9	−6.6	3.579	5.289
Analog-5 RMSD values of different poses against alpha-glucosidase			

**TABLE 3:** RMSD values of different poses of analog 9d against α-amylase and α-glucosidase enzymes.

Mode	Affinity	Distance from best mode	
	(kcal/mol)	rmsd I.b	rmsd u.b
1	−8.5	0.000	0.000
2	−8.5	2.246	3.435
3	−8.1	3.338	5.131
4	−8.0	3.707	6.835
5	−8.0	3.020	6.097
6	−7.9	2.451	3.725
7	−7.9	3.255	5.056
8	−7.9	2.376	3.374
9	−7.9	5.022	7.801
Analog-5 RMSD values of different poses against alpha-amylase			

Mode	Affinity	Distance from best mode	
	(kcal/mol)	rmsd I.b	rmsd u.b
1	−8.6	0.000	0.000
2	−7.6	1.389	1.693
3	−7.5	2.502	3.433
4	−7.4	2.592	3.214
5	−7.3	8.077	9.946
6	−7.0	2.896	3.851
7	−6.9	8.501	10.238
8	−6.7	3.089	5.590
9	−6.7	3.919	6.462
Analog-5 RMSD values of different poses against alpha-glucosidase			

**TABLE 4:** RMSD values of different poses of standard acarbose drug against α-amylase and α-glucosidase enzymes.

Mode	Affinity	Distance from best mode	
	(kcal/mol)	rmsd I.b	rmsd u.b
1	−8.0	0.000	0.000
2	−7.8	3.267	11.500
3	−7.8	3.228	10.552
4	−7.7	2.163	9.832
5	−7.5	4.169	7.220
6	−7.5	5.320	9.443
7	−7.4	4.477	8.490
8	−7.4	3.580	11.779
9	−7.2	5.259	9.041
Acarbose RMSD values against alpha-amylase			

Mode	Affinity	Distance from best mode	
	(kcal/mol)	rmsd I.b	rmsd u.b
1	−7.3	0.000	0.000
2	−7.3	3.562	11.099
3	−7.3	1.489	2.269
4	−6.9	3.817	10.685
5	−6.9	3.838	10.852
6	−6.7	7.042	13.565
7	−6.7	6.866	11.671
8	−6.6	2.089	11.269
9	−6.6	4.540	11.478
Acarbose RMSD values against alpha-glucosidase			

## 4 Conclusion

In conclusion, the present study synthesized novel hybrid analogs **(1d–19d)** based on a quinoline-bearing triazole moiety in their core structure. The entire constructed analogs were also additionally assessed for α-amylase and α-glucosidase (*in vitro*) inhibitory activities. All scaffolds being synthesized were found to display a diverse range of inhibitory potentials between 0.80 ± 0.05µM and 40.20 ± 0.70 µM (against α-amylase) and 1.20 ± 0.10 µM and 43.30 ± 0.80 µM (against α-glucosidase). Compound **11d** (IC_50_ = 0.80 ± 0.05 µM) (IC_50_ = 1.20 ± 0.10 µM) holding *para*-fluoro substitution on the phenyl ring emerged as the most active analog among the synthesized series. Moreover, compound **9d** with fluoro substitution at the *ortho*-position of the phenyl ring was identified as the second most active analog against α-amylase and α-glucosidase enzymes. Therefore, it was concluded based on SAR studies that scaffolds bearing electron-withdrawing groups such as–NO_2_, -Cl, and –F attached to the phenyl ring, resulting in enhanced inhibitory potentials. When these active scaffolds **11d** and **9d** underwent molecular docking analysis, they created a number of significant contacts with the active sites of the two targeted enzymes. The protein–ligand contact profile for compound 11d against α-amylase shows that it established numerous important interactions such as His-305 (donor interactions), Tyr-62 (π–π stacked), His-299 (donor interactions), Asp-3002 (H-B), Glu-233 (H-B), Leu-162 (π–R), and Trp-59 (π–π T-shaped), while against α-glucosidase it also furnishes numerous important interactions, including Met-470 (π-S), Ala-234 (π–R), Asp-568 (π–anion), Trp-329 (π–π T-shaped), Trp-432 (π–sigma), Arg-552 (H-B), and Asp-232 (π–anion). Furthermore, the structures of all the newly synthesized quinoline-based triazole analogs were confirmed by employing different techniques, including ^1^H NMR, HREI-MS, and ^13^C-NMRspectroscopy.

## Data Availability

The original contributions presented in the study are included in the article/Supplementary Material; further inquiries can be directed to the corresponding authors.
