# Supplementary material for: New quinoline-based triazole hybrid analogs as effective inhibitors of α-amylase and α-glucosidase: Preparation, in vitro evaluation, and molecular docking along with in silico studies
Source: Front Chem. 2022 Sep 15;10:995820. doi: 10.3389/fchem.2022.995820 (PMC9520911; doi:10.3389/fchem.2022.995820)

**Supporting Information**

**New Quinoline-based triazole hybrid analogues as effective inhibitors of α-amylase and α-glucosidase: preparation, *in vitro* evaluation and molecular docking along with *in silico* studies**

Yousaf Khan^a^, Shahid Iqbal^b*^, Mazloom Shah^c^, Aneela Maalik^a^, Rafaqat Hussain^d^, Shoaib Khan^d*^, Imran Khan^d^, Rami Adel Pashameah^e^, Eman Alzahrani^f^, Abd-El AziemFarouk^g^, Mohammed Issa Alahmdi^h^, Hisham S.M. Abd-Rabboh^i,j^

*^a^Department of Chemistry, COMSATS Universityislamabad campus-45550, islamabad, Pakistan.*

*^b^Department of Chemistry, School of Natural Sciences (SNS), National University of Science and Technology (NUST), H-12, Islamabad, 46000, Pakistan.*

*^c^Department of Chemistry, Abbottabad University of Science and Technology (AUST) Abbottabad, Pakistan.*

*^d^Department of Chemistry, Hazara University, Mansehra-21120, Pakistan.*

*^e^Department of Chemistry, Faculty of Applied Science, Umm Al-Qura University, Makkah 24230, Saudi Arabia.*

*^f^Department of Chemistry, College of Science, Taif University, P.O. Box 11099, Taif 21944, Saudi Arabia.*

*^g^Department of Biotechnology College of Science, Taif University, P.O. Box 11099, Taif 21944, Saudi Arabia.*

*^h^Department of Chemistry, Faculty of Science, University of Tabuk, Tabuk- 71491, Saudi Arabia.*

*^i^Chemistry Department, Faculty of Science, King Khalid University, P.O. Box 9004, Abha 61413, Saudi Arabia.*

*^j^Department of Chemistry, Faculty of Science, Ain Shams University, Abbassia, Cairo 11566, Egypt.*

****To whom corresponding should be addressed***

[shahidgcs10@yahoo.com](mailto:shahidgcs10@yahoo.com)(Shahid Iqbal) and shoaibkhanswati@gmail.com (Shoaib Khan)

**2.2. Experimental**

**General procedure for affording quinoline-based triazole scaffolds (1d-19d):**

Quinoline-based triazole derivatives were afforded in 3 steps: initially, methyl quinoline-7-carboxylate (0.5mmol) **(a)** was treated N_2_H_4_.H_2_O (1 equivalent) in EtOH (10mL) and stirred under reflux for 5h to afford quinoline-7-carbohydrazide (0.5mmol) as an intermediate **(b)**. In the second step, an intermediate **(b)** was further treated with corresponding phenyl isothiocyanates (1 equivalent) and Et_3_N (1mL) in THF (10mL), and the resulting mixture was refluxed for 12h to obtain substrate **(c)** which further underwent cyclization with 2% NaOH (10mL) followed by neutralization with dil HCl (5mL) access the formation of the targeted quinoline based triazole **(1d-19d)** analogues in good to excellent yield. Synthesized compounds **(1d-19d)** were characterized to explore the ^1^H and ^13^C and HREI-MS. Different substituted compounds showed a varied range of coupling due to electron withdrawing and donating effects of attached substituents. The characterized analogs are:

**2.2.1. 4-Phenyl-5-(quinolin-7-yl)-2,4-dihydro-3H-1,2,4-triazole-3-thione (1d)**

Yield: 55 % (0.30 gm), M.P (^o^C): 137-138, brown solid; ^1^H-NMR (500MHz, DMSO-*d*_6_): *δ* 12.00 (s, NH, 1H), 8.66 (s, Quinoline-H, 1H), 8.61 (dd, *J =* 7.7, 1.4 Hz*,* Quinoline-H, 1H), 8.59 (d, *J =* 8.1 Hz*,* Quinoline-H, 1H), 8.51 (dd, *J =* 6.9, 2.0 Hz*,* Quinoline-H, 1H), 8.42 (d, *J =* 7.6 Hz*,* Quinoline-H, 1H), 7.60 (t, *J =* 7.1 Hz*,* Quinoline-H, 1H), 7.30 (dd, *J =* 7.9, 2.1 Hz*,* Aroma-H, 2H), 7.16 (t, *J* = 7.6 Hz, Aroma-H, 1H), 7.14-7.08 (m, Aroma-H, 2H); ^13^C-NMR (125MHz, DMSO-*d*_6_): *δ* 166.0, 151.1, 148.3, 141.2, 140.5, 139.0, 137.3, 136.4, 135.6, 133.7, 129.8, 127.1, 127.0, 125.5, 124.4, 122.2, 121.6; HR-EIMS: *m/z* calc. for C_17_H_12_N_4_S [M]^+^ 304.0667; Found 304.0645.

**2.2.2. 5-(Quinolin-7-yl)-4-(o-tolyl)-2,4-dihydro-3H-1,2,4-triazole-3-thione (2d)**

Yield: 60 % (0.33 gm), M.P (^o^C): 139-140, white solid; ^1^H-NMR (500MHz, DMSO-*d*_6_): *δ* 12.03 (s, NH, 1H), 8.73 (s, Quinoline-H, 1H), 8.71 (dd, *J =* 8.0, 1.4 Hz*,* Quinoline-H, 1H), 8.67 (d, *J =* 7.8 Hz*,* Quinoline-H, 1H), 8.58 (dd, *J =* 7.3, 2.1 Hz*,* Quinoline-H, 1H), 8.48 (d, *J =* 7.6 Hz*,* Quinoline-H, 1H), 7.77 (t, *J =* 7.1 Hz*,* Quinoline-H, 1H), 7.49 (dd, *J =* 7.6, 2.5 Hz*,* Aroma-H, 1H), 7.45-7.41 (m, Aroma-H, 1H); 7.39-7.35 (m, Aroma-H, 1H),7.30 (dd, *J* = 7.6, 1.6 Hz, Aroma-H, 1H), 2.40 (s, CH_3_, 3H); ^13^C-NMR (125MHz, DMSO-*d*_6_): *δ* 167.6, 151.0, 148.1, 141.7, 140.6, 139.3, 137.2, 136.4, 135.9, 133.7, 129.8, 127.3, 127.2, 126.2, 125.1, 124.0, 122.4, 46.7; HR-EIMS: *m/z* calc. for C_18_H_14_N_4_S [M]^+^ 318.0727; Found 318.0710.

**2.2.3. 5-(Quinolin-7-yl)-4-(m-tolyl)-2,4-dihydro-3H-1,2,4-triazole-3-thione (3d)**

Yield: 54 % (0.29 gm), M.P (^o^C): 136-137, white solid; ^1^H-NMR (500MHz, DMSO-*d*_6_): *δ* 12.04 (s, NH, 1H), 8.74 (s, Quinoline-H, 1H), 8.70 (dd, *J =* 7.0, 1.3 Hz*,* Quinoline-H, 1H), 8.65 (d, *J =* 8.0 Hz*,* Quinoline-H, 1H), 8.59 (dd, *J =* 7.1, 2.2 Hz*,* Quinoline-H, 1H), 8.49 (d, *J =* 7.6 Hz*,* Quinoline-H, 1H), 7.79 (t, *J =* 7.3 Hz*,* Quinoline-H, 1H), 7.51 (dd, *J =* 7.4, 2.3 Hz*,* Aroma-H, 1H), 7.45 (s, Aroma-H, 1H), 7.41 (t, *J* = 7.7 Hz, Aroma-H, 1H), 7.36 (dd, *J* = 7.1, 1.8 Hz, Aroma-H, 2H), 2.43 (s, CH_3_, 3H); ^13^C-NMR (125MHz, DMSO-*d*_6_): *δ* 167.3, 151.4, 148.2, 141.6, 140.9, 139.1, 137.6, 136.1, 135.1, 133.9, 129.7, 127.4, 127.0, 126.5, 125.4, 124.2, 122.1, 46.0; HR-EIMS: *m/z* calc. for C_18_H_14_N_4_S [M]^+^ 318.0747; Found 318.0730.

**2.2.4. 5-(Quinolin-7-yl)-4-(p-tolyl)-2,4-dihydro-3H-1,2,4-triazole-3-thione (4d)**

Yield: 52 % (0.27 gm), M.P (^o^C): 127-128, white solid; ^1^H-NMR (500MHz, DMSO-*d*_6_): *δ* 12.03 (s, NH, 1H), 8.72 (s, Quinoline-H, 1H), 8.69 (dd, *J =* 8.0, 1.3 Hz*,* Quinoline-H, 1H), 8.63 (d, *J =* 7.8 Hz*,* Quinoline-H, 1H), 8.52 (dd, *J =* 7.3, 2.0 Hz*,* Quinoline-H, 1H), 8.45 (d, *J =* 7.7 Hz*,* Quinoline-H, 1H), 7.76 (t, *J =* 8.0 Hz*,* Quinoline-H, 1H), 7.48 (d, *J =* 7.4 Hz*,* Aroma-H, 2H), 7.39 (d, *J* = 7.1 Hz, Aroma-H, 2H), 2.40 (s, CH_3_, 3H); ^13^C-NMR (125MHz, DMSO-*d*_6_): *δ* 167.1, 151.5, 148.5, 141.5, 140.8, 139.1, 137.6, 136.1, 135.1, 133.9, 129.7, 127.2, 127.2, 126.1, 125.7, 124.9, 122.1, 46.1; HR-EIMS: *m/z* calc. for C_18_H_14_N_4_S [M]^+^ 318.0777; Found 318.0740.

**2.2.5. 4-(2-Nitrophenyl)-5-(quinolin-7-yl)-2,4-dihydro-3H-1,2,4-triazole-3-thione (5d)**

Yield: 70 % (0.39 gm), M.P (^o^C): 139-140, light yellow solid; ^1^H-NMR (500MHz, DMSO-*d*_6_): *δ* 12.02 (s, NH, 1H), 8.70 (s, Quinoline-H, 1H), 8.67 (dd, *J =* 7.0, 1.3 Hz*,* Quinoline-H, 1H), 8.63 (d, *J =* 7.6 Hz*,* Quinoline-H, 1H), 8.59 (dd, *J =* 7.3, 2.0 Hz*,* Quinoline-H, 1H), 8.44 (d, *J =* 7.4 Hz*,* Quinoline-H, 1H), 7.73 (t, *J =* 6.8 Hz*,* Quinoline-H, 1H), 7.49 (dd, *J =* 7.5, 1.8 Hz*,* Aroma-H, 1H), 7.44-7.40 (m, Aroma-H, 1H), 7.39-7.33 (m, Aroma-H, 1H),7.30 (dd, *J =* 7.2, 1.8 Hz*,* Aroma-H, 1H); ^13^C-NMR (125MHz, DMSO-*d*_6_): *δ* 169.4, 151.6, 148.7, 142.6, 141.5, 138.2, 136.5, 135.2, 135.1, 134.8, 130.0, 128.2, 128.0, 127.3, 126.4, 124.5, 122.2; HR-EIMS: *m/z* calc. for C_17_H_11_N_5_O_2_S [M]^+^ 349.0430; Found 349.0411.

**2.2.6. 4-(3-Nitrophenyl)-5-(quinolin-7-yl)-2,4-dihydro-3H-1,2,4-triazole-3-thione (6d)**

Yield: 69 % (0.38 gm), M.P (^o^C): 130-131, yellow solid; ^1^H-NMR (500MHz, DMSO-*d*_6_): *δ*12.03 (s, NH, 1H), 8.71 (s, Quinoline-H, 1H), 8.64 (dd, *J =* 7.8, 1.4 Hz*,* Quinoline-H, 1H), 8.60 (d, *J =* 7.6 Hz*,* Quinoline-H, 1H), 8.56 (dd, *J =* 7.7, 2.1 Hz*,* Quinoline-H, 1H), 8.41 (d, *J =* 7.0 Hz*,* Quinoline-H, 1H), 7.70 (t, *J =* 6.9 Hz*,* Quinoline-H, 1H), 7.46 (dd, *J =* 7.6, 1.9 Hz*,* Aroma-H, 1H), 7.40 (s, Aroma-H, 1H), 7.36 (t, *J* = 8.0Hz, Aroma-H, 1H), 7.25 (dd, *J =* 7.7, 1.3 Hz*,* Aroma-H, 1H); ^13^C-NMR (125MHz, DMSO-*d*_6_): *δ* 169.3, 151.5, 148.6, 142.5, 141.4, 138.1, 136.4, 135.0, 135.1, 134.8, 130.9, 128.8, 128.7, 127.2, 126.3, 124.4, 122.1; HR-EIMS: *m/z* calc. for C_17_H_11_N_5_O_2_S [M]^+^ 349.0426; Found 349.0406.

**2.2.7. 4-(4-Nitrophenyl)-5-(quinolin-7-yl)-2,4-dihydro-3H-1,2,4-triazole-3-thione (7d)**

Yield: 67 % (0.36 gm), M.P (^o^C): 127-128, dark yellow solid; ^1^H-NMR (500MHz, DMSO-*d*_6_): *δ* 12.00 (s, NH, 1H), 8.70 (s, Quinoline-H, 1H), 8.62 (dd, *J =* 8.0, 1.4 Hz*,* Quinoline-H, 1H), 8.62 (d, *J =* 7.7 Hz*,* Quinoline-H, 1H), 8.54 (dd, *J =* 7.1, 2.1 Hz*,* Quinoline-H, 1H), 8.45 (d, *J =* 7.3 Hz*,* Quinoline-H, 1H), 7.71 (t, *J =* 6.9 Hz*,* Quinoline-H, 1H), 7.47 (d, *J =* 7.1 Hz*,* Aroma-H, 2H), 7.39 (d, *J* = 7.7 Hz, Aroma-H, 2H); ^13^C-NMR (125MHz, DMSO-*d*_6_): *δ* 169.1, 151.5, 148.5, 142.5, 141.8, 138.1, 136.6, 135.1, 135.1, 134.9, 130.7, 128.4, 128.1, 127.1, 126.7, 124.2, 122.1; HR-EIMS: *m/z* calc. for C_17_H_11_N_5_O_2_S [M]^+^ 349.0450; Found 349.0420.

**2.2.8. 4-(4-Methoxyphenyl)-5-(quinolin-7-yl)-2,4-dihydro-3H-1,2,4-triazole-3-thione (8d)**

Yield: 56 % (0.30 gm), M.P (^o^C): 134-135, white solid; ^1^H-NMR (500MHz, DMSO-*d*_6_): *δ*12.01 (s, NH, 1H), 8.71 (s, Quinoline-H, 1H), 8.63 (dd, *J =* 8.1, 1.4 Hz*,* Quinoline-H, 1H), 8.60 (d, *J =* 7.9 Hz*,* Quinoline-H, 1H), 8.50 (dd, *J =* 7.0, 2.1 Hz*,* Quinoline-H, 1H), 8.43 (d, *J =* 7.8 Hz*,* Quinoline-H, 1H), 7.72 (t, *J =* 8.2 Hz*,* Quinoline-H, 1H), 7.42 (d, *J =* 7.5 Hz*,* Aroma-H, 2H), 7.34 (d, *J* = 7.5 Hz, Aroma-H, 2H), 2.45 (s, CH_3_, 3H); ^13^C-NMR (125MHz, DMSO-*d*_6_): *δ* 168.1, 150.5, 147.5, 141.5, 140.8, 137.1, 135.6, 134.1, 134.1, 133.9, 129.7, 127.4, 127.1, 126.1, 125.7, 124.2, 122.1, 46.7; HR-EIMS: *m/z* calc. for C_18_H_14_N_4_OS [M]^+^ 334.0750; Found 334.0720.

**2.2.9. 4-(2-Fluorophenyl)-5-(quinolin-7-yl)-2,4-dihydro-3H-1,2,4-triazole-3-thione (9d)**

Yield: 62 % (0.35 gm), M.P (^o^C): 129-130, Reddish brown solid;^1^H-NMR (500MHz, DMSO-*d*_6_): *δ*12.07 (s, NH, 1H), 8.79 (s, Quinoline-H, 1H), 8.70 (dd, *J =* 7.5, 2.3 Hz*,* Quinoline-H, 1H), 8.69 (d, *J =* 7.7 Hz*,* Quinoline-H, 1H), 8.65 (dd, *J =* 7.1, 1.8 Hz*,* Quinoline-H, 1H), 8.51 (d, *J =* 7.7 Hz*,* Quinoline-H, 1H), 7.80 (t, *J =* 8.2 Hz*,* Quinoline-H, 1H), 7.55 (dd, *J =* 7.1, 2.4 Hz*,* Aroma-H, 1H), 7.49-7.45 (m, Aroma-H, 1H), 7.43-7.38 (m, Aroma-H, 1H), 7.35 (dd, *J* = 8.0, 2.1 Hz, Aroma-H, 1H); ^13^C-NMR (125MHz, DMSO-*d*_6_): *δ* 169.8, 149.7, 146.4, 140.3, 139.7, 136.0, 134.5, 134.1, 134.4, 133.1, 129.9, 128.6, 127.2, 126.2, 125.1, 124.7, 123.2; HR-EIMS: *m/z* calc. for C_17_H_11_FN_4_S [M]^+^ 322.0601; Found 322.0588.

**2.2.10. 4-(3-Fluorophenyl)-5-(quinolin-7-yl)-2,4-dihydro-3H-1,2,4-triazole-3-thione (10d)**

Yield: 63 % (0.35 gm), M.P (^o^C): 128-129, reddish brown solid; ^1^H-NMR (500MHz, DMSO-*d*_6_): *δ* 12.06 (s, NH, 1H), 8.76 (s, Quinoline-H, 1H), 8.67 (dd, *J =* 7.4, 1.9 Hz*,* Quinoline-H, 1H), 8.65 (d, *J =* 7.8 Hz*,* Quinoline-H, 1H), 8.57 (dd, *J =* 7.3, 1.6 Hz*,* Quinoline-H, 1H), 8.44 (d, *J =* 7.8 Hz*,* Quinoline-H, 1H), 7.76 (t, *J =* 8.1 Hz*,* Quinoline-H, 1H), 7.51 (dd, *J =* 7.5, 2.1 Hz*,* Aroma-H, 1H), 7.49 (s, Aroma-H, 1H), 7.40 (t, *J* = 7.3 Hz, Aroma-H, 1H), 7.25 (dd, *J* = 7.1, 2.3 Hz, Aroma-H, 1H); ^13^C-NMR (125MHz, DMSO-*d*_6_): *δ* 166.8, 149.9, 146.5, 140.6, 139.8, 136.2, 134.6, 134.3, 134.2, 133.1, 129.8, 128.5, 127.4, 126.0, 125.3, 124.0, 123.3; HR-EIMS: *m/z* calc. for C_17_H_11_FN_4_S [M]^+^ 322.0520; Found 322.0508.

**2.2.11. 4-(4-Fluorophenyl)-5-(quinolin-7-yl)-2,4-dihydro-3H-1,2,4-triazole-3-thione (11d)**

Yield: 65 % (0.34 gm), M.P (^o^C): 131-132, reddish brown solid; ^1^H-NMR (500MHz, DMSO-*d*_6_): *δ*12.03 (s, NH, 1H), 8.73 (s, Quinoline-H, 1H), 8.64 (dd, *J =* 8.4, 1.3 Hz*,* Quinoline-H, 1H), 8.61 (d, *J =* 7.2 Hz*,* Quinoline-H, 1H), 8.53 (dd, *J =* 7.1, 2.3 Hz*,* Quinoline-H, 1H), 8.41 (d, *J =* 7.1 Hz*,* Quinoline-H, 1H), 7.74 (t, *J =* 8.0 Hz*,* Quinoline-H, 1H), 7.46 (d, *J =* 7.6 Hz*,* Aroma-H, 2H), 7.36 (d, *J* = 7.9 Hz, Aroma-H, 2H); ^13^C-NMR (125MHz, DMSO-*d*_6_): *δ* 167.1, 149.5, 146.5, 140.5, 139.8, 136.1, 134.6, 134.2, 134.2, 133.0, 129.6, 128.4, 127.3, 126.1, 125.7, 124.2, 123.1; HR-EIMS: *m/z* calc. for C_17_H_11_FN_4_S [M]^+^ 322.0420; Found 322.0412.

**2.2.12. 4-(2-Chlorophenyl)-5-(quinolin-7-yl)-2,4-dihydro-3H-1,2,4-triazole-3-thione (12d)**

Yield: 67 % (0.37 gm), M.P (^o^C): 136-137, white solid; ^1^H-NMR (500MHz, DMSO-*d*_6_): *δ* 11.97 (s, NH, 1H), 8.66 (s, Quinoline-H, 1H), 8.61 (dd, *J =* 8.0, 1.5 Hz*,* Quinoline-H, 1H), 8.60 (d, *J =* 7.6 Hz*,* Quinoline-H, 1H), 8.56 (dd, *J =* 8.3, 2.3 Hz*,* Quinoline-H, 1H), 8.46 (d, *J =* 7.9 Hz*,* Quinoline-H, 1H), 7.72 (t, *J =* 7.4 Hz*,* Quinoline-H, 1H), 7.48 (dd, *J =* 7.4, 1.8 Hz*,* Aroma-H, 1H), 7.45-7.40 (m, Aroma-H, 1H), 7.38-7.31 (m, Aroma-H, 1H), 7.26 (dd, *J* = 7.2, 2.0 Hz, Aroma-H, 1H); ^13^C-NMR (125MHz, DMSO-*d*_6_): *δ* 168.0, 150.2, 148.5, 142.2, 140.7, 137.2, 136.6, 134.7, 134.1, 133.8, 132.6, 130.9, 129.1, 128.0, 127.6, 126.1, 125.7; HR-EIMS: *m/z* calc. for C_17_H_11_ClN_4_S [M]^+^ 338.0510; Found 338.0496.

**2.2.13. 4-(3-Chlorophenyl)-5-(quinolin-7-yl)-2,4-dihydro-3H-1,2,4-triazole-3-thione (13d)**

Yield: 63 % (0.34 gm), M.P (^o^C): 141-142, white solid; ^1^H-NMR (500MHz, DMSO- *d*_6_): *δ* 11.95 (s, NH, 1H), 8.73 (s, Quinoline-H, 1H), 8.69 (dd, *J =* 8.0, 1.5 Hz*,* Quinoline-H, 1H), 8.69 (d, *J =* 7.9 Hz*,* Quinoline-H, 1H), 8.59 (dd, *J =* 7.3, 2.3 Hz*,* Quinoline-H, 1H), 8.50 (d, *J =* 7.6 Hz*,* Quinoline-H, 1H), 7.76 (t, *J =* 7.9 Hz*,* Quinoline-H, 1H), 7.46 (dd, *J =* 7.6, 2.1 Hz*,* Aroma-H, 1H), 7.40 (t, *J* = 7.7 Hz, Aroma-H, 1H), 7.33 (s, Aroma-H, 1H), 7.25 (dd, *J* = 7.2, 1.9 Hz, Aroma-H, 1H); ^13^C-NMR (125MHz, DMSO-*d*_6_): *δ* 168.3, 150.4, 148.6, 142.3, 140.8, 137.4, 136.5, 134.4, 134.1, 133.8, 132.6, 130.9, 129.1, 128.0, 127.2, 126.3, 125.4; HR-EIMS: *m/z* calc. for C_17_H_11_ClN_4_S [M]^+^ 338.0530; Found 338.0515.

**2.2.14. 4-(4-Chlorophenyl)-5-(quinolin-7-yl)-2,4-dihydro-3H-1,2,4-triazole-3-thione (14d)**

Yield: 66 % (0.35 gm), M.P (^o^C): 131-132, white solid; ^1^H-NMR (500MHz, DMSO-*d*_6_): *δ* 11.96 (s, NH, 1H), 8.65 (s, Quinoline-H, 1H), 8.60 (dd, *J =* 7.4, 1.5 Hz*,* Quinoline-H, 1H), 8.59 (d, *J =* 7.7 Hz*,* Quinoline-H, 1H), 8.55 (dd, *J =* 7.5, 2.1 Hz*,* Quinoline-H, 1H), 8.45 (d, *J =* 7.6 Hz*,* Quinoline-H, 1H), 7.71 (t, *J =* 7.9 Hz*,* Quinoline-H, 1H), 7.47 (d, *J =* 7.3 Hz*,* Aroma-H, 2H), 7.36 (d, *J* = 8.1 Hz, Aroma-H, 2H); ^13^C-NMR (125MHz, DMSO-*d*_6_): *δ* 167.2, 149.3, 147.4, 141.4, 140.7, 137.0, 135.5, 134.4, 134.4, 133.2, 130.5, 129.3, 128.2, 127.0, 126.6, 125.1, 124.0; HR-EIMS: *m/z* calc. for C_17_H_11_ClN_4_S [M]^+^ 338.0520; Found 338.0504.

**2.2.15. 4-(2-Bromophenyl)-5-(quinolin-7-yl)-2,4-dihydro-3H-1,2,4-triazole-3-thione (15d)**

Yield: 59 % (0.32 gm), M.P (^o^C): 135-136, white solid; ^1^H-NMR (500MHz, DMSO-*d*_6_): *δ* 12.00 (s, NH, 1H), 8.79 (s, Quinoline-H, 1H), 8.71 (dd, *J =* 8.1, 1.4 Hz*,* Quinoline-H, 1H), 8.68 (d, *J =* 7.7 Hz*,* Quinoline-H, 1H), 8.63 (dd, *J =* 7.3, 2.1 Hz*,* Quinoline-H, 1H), 8.55 (d, *J =* 7.5 Hz*,* Quinoline-H, 1H), 7.89 (t, *J =* 7.4 Hz*,* Quinoline-H, 1H), 7.70 (dd, *J =* 7.5, 2.1 Hz*,* Aroma-H, 1H), 7.65-7.60 (m, Aroma-H, 1H), 7.55-7.50 (m, Aroma-H, 1H), 7.45 (dd, *J* = 7.1, 2.1 Hz, Aroma-H, 1H); ^13^C-NMR (125MHz, DMSO-*d*_6_): *δ* 168.1, 150.3, 148.4, 142.2, 140.7, 137.2, 136.5, 134.3, 134.1, 133.2, 132.4, 130.0, 129.2, 128.0, 127.6, 126.1, 125.8; HR-EIMS: *m/z* calc. for C_17_H_11_BrN_4_S [M]^+^ 381.7725; Found 381.7717.

**2.2.16. 4-(3-Bromophenyl)-5-(quinolin-7-yl)-2,4-dihydro-3H-1,2,4-triazole-3-thione (16d)**

Yield: 57 % (0.31 gm), M.P (^o^C): 130-131, white solid; ^1^H-NMR (500MHz, DMSO-*d*_6_): *δ* 11.99 (s, NH, 1H), 8.72 (s, Quinoline-H, 1H), 8.68 (dd, *J =* 8.2, 1.5 Hz*,* Quinoline-H, 1H), 8.66 (d, *J =* 7.8 Hz*,* Quinoline-H, 1H), 8.58 (dd, *J =* 7.1, 2.0 Hz*,* Quinoline-H, 1H), 8.48 (d, *J =* 7.5 Hz*,* Quinoline-H, 1H), 7.78 (t, *J =* 7.6 Hz*,* Quinoline-H, 1H), 7.47 (dd, *J =* 7.6, 2.1 Hz*,* Aroma-H, 1H), 7.41 (t, *J* = 7.0 Hz, Aroma-H, 1H), 7.33 (s, Aroma-H, 1H), 7.27 (dd, *J* = 7.3, 2.1 Hz, Aroma-H, 1H); ^13^C-NMR (125MHz, DMSO-*d*_6_): *δ* 167.1, 149.3, 147.4, 141.2, 140.7, 137.2, 135.5, 134.3, 134.1, 133.2, 130.4, 129.0, 128.2, 127.0, 126.6, 125.1, 124.8; HR-EIMS: *m/z* calc. for C_17_H_11_BrN_4_S [M]^+^ 381.7820; Found 381.7812.

**2.2.17. 4-(4-Bromophenyl)-5-(quinolin-7-yl)-2,4-dihydro-3H-1,2,4-triazole-3-thione (17d)**

Yield: 54 % (0.29 gm), M.P (^o^C): 132-133, reddish brown solid; ^1^H-NMR (500MHz, DMSO-*d*_6_): *δ* 11.98 (s, NH, 1H), 8.70 (s, Quinoline-H, 1H), 8.67 (dd, *J =* 8.1, 1.4 Hz*,* Quinoline-H, 1H), 8.65 (d, *J =* 7.7 Hz*,* Quinoline-H, 1H), 8.57 (dd, *J =* 7.3, 2.2 Hz*,* Quinoline-H, 1H), 8.47 (d, *J =* 7.4 Hz*,* Quinoline-H, 1H), 7.77 (t, *J =* 7.9 Hz*,* Quinoline-H, 1H), 7.49 (d, *J =* 7.6 Hz*,* Aroma-H, 2H), 7.38 (d, *J* = 7.1 Hz, Aroma-H, 2H); ^13^C-NMR (125MHz, DMSO-*d*_6_): *δ* 167.3, 149.4, 147.5, 141.5, 140.8, 137.1, 135.6, 134.5, 134.5, 133.3, 130.6, 129.4, 128.3, 127.1, 126.7, 125.2, 124.1; HR-EIMS: *m/z* calc. for C_17_H_11_BrN_4_S [M]^+^ 381.7920; Found 381.7612.

**2.2.18. 4-(3,4-Dichlorophenyl)-5-(quinolin-7-yl)-2,4-dihydro-3H-1,2,4-triazole-3-thione (18d)**

Yield: 69 % (0.38 gm), M.P (^o^C): 133-134, white solid; ^1^H-NMR (500MHz, DMSO-*d*_6_): *δ* 12.04 (s, NH, 1H), 8.74 (s, Quinoline-H, 1H), 8.68 (dd, *J =* 7.3, 1.4 Hz*,* Quinoline-H, 1H), 8.65 (d, *J =* 7.9 Hz*,* Quinoline-H, 1H), 8.55 (dd, *J =* 7.6, 1.7 Hz*,* Quinoline-H, 1H), 8.43 (d, *J =* 7.1 Hz*,* Quinoline-H, 1H), 7.74 (t, *J =* 8.0 Hz*,* Quinoline-H, 1H), 7.50 (d, *J =* 7.6 Hz*,* Aroma-H, 1H), 7.47 (s, Aroma-H, 1H), 7.23 (d, *J* = 7.2 Hz, Aroma-H, 1H); ^13^C-NMR (125MHz, DMSO-*d*_6_): *δ* 166.4, 149.7, 146.2, 140.7, 139.4, 136.0, 134.7, 134.8, 134.1, 133.4, 129.6, 128.7, 127.5, 126.6, 125.3, 124.0, 123.5; HR-EIMS: *m/z* calc. for C_17_H_10_Cl_2_N_4_S [M]^+^ 372.0120; Found 372.0090. **2.2.19. 4-([1,1'-Biphenyl]-4-yl)-5-(quinolin-7-yl)-2,4-dihydro-3H-1,2,4-triazole-3-thione (19d)**

Yield: 55 % (0.28 gm), M.P (^o^C): 138-139, white solid; ^1^H-NMR (500MHz, DMSO-*d*_6_): *δ* 12.10 (s, NH, 1H), 8.77 (s, Quinoline-H, 1H), 8.71 (dd, *J =* 7.4, 1.5 Hz*,* Quinoline-H, 1H), 8.69 (d, *J =* 7.6 Hz*,* Quinoline-H, 1H), 8.59 (dd, *J =* 7.9, 1.4 Hz*,* Quinoline-H, 1H), 8.41 (d, *J =* 7.2Hz*,* Quinoline-H, 1H), 7.77 (t, *J =* 8.2Hz*,* Quinoline-H, 1H), 7.61 (dd, *J =* 7.8, 1.8 Hz*,* Aroma-H, 2H), 7.55 (d, *J =* 7.7Hz*,* Aroma-H, 2H), 7.52-7.47 (m, Aroma-H, 1H), 7.39 (t, *J* = 6.9 Hz, Aroma-H, 1H), 7.28 (d, *J* = 7.9 Hz, Aroma-H, 2H); ^13^C-NMR (125MHz, DMSO-*d*_6_): *δ* 166.5, 149.6, 146.3, 140.2, 139.7, 136.1, 134.2, 134.0, 134.0, 133.5, 129.9, 128.8, 128.8, 128.6, 128.0, 128.0, 127.5, 127.5, 127.3, 126.3, 124.1, 121.0; HR-EIMS: *m/z* calc. for C_23_H_16_N_4_S [M]^+^ 380.0980; Found 380.0870.

**4.1. *α*-Glucosidase assay protocol**

70 μL 50 mM phosphate buffer pH 6.8 and 10 μL (0.5 mM in methanol) test analogue were mixed to afford a total volume of 100 μL mixture, after that 10 μL (0.057 units, Sigma Inc.) enzyme solution in the buffer solution was added to it. The contents were pre-incubated for 10 min at 37 °C and further mixed and pre-read at 400 nm. The 10 μL of 0.5 mM substrate (p-nitrophenyl glucopyranoside, Sigma Inc.) was added to the reaction to initiate the reaction. Using the Synergy HT 96-well plate reader, BioTek, USA, the absorbance of p-nitrophenol was measured at 400 nm after 30 min of incubation at 37 °C under the positive control of Acarbose. The triplicates (mean ± SEM, n = 3) were employed to carry out all experiments. The following equation: Inhibition (%) = (Abs of Control-Abs of Test/Abs of Control) ×100 was used to calculate the percent inhibition through dilution of the solution of active scaffolds. By using EZ-Fit Enzyme Kinetics Software (Perrella Scientific Inc. Amherst, USA), the data obtained was used for the determination of IC_50_ values (concentration at which there is 50 % enzyme inhibition).

**4.2. *α*-Amlyase inhibition assay**

The synthetic compounds were dissolved in DMSO to obtain concentrations of 10, 20, 40, 60, 80, and 100 μg/mL A total of 40 μL of sample and 40 μL of 0.02 M sodium phosphate buffer (pH 6.9 with 0.006 M sodium chloride) containing α- amylase solution (Porcine pancreatic α- amylase) (0.5 mg/ml) were incubated at 25 °C for 10 min. After preincubation, 40 μL of a 1% starch solution in 0.02 M sodium phosphate buffer (pH 6.9 with 0.006 M sodium chloride) was added to each tube at 5 s intervals. The reaction mixtures were then incubated at 25 °C for 10 min. The reaction was stopped with 100 μL of dinitro salicylic acid color reagent. The test tubes were then incubated in a boiling water bath for 5 min and cooled to room temperature. The reaction mixture was then diluted after adding 900 μL distilled water and the absorbance was measured at 540 nm. Calculation of the concentration of compound required to scavenge 50% of the radical (IC_50_) as per the formula below: I% = (Ac-As)/Ac x 100

Ac = the absorbance of the control

As = the absorbance of the sample

**4.3. Molecular docking assay**

A molecular docking study was conducted by using discovery studio visualizer (DSV) MGL tool 1.5.7 and auto Dock vina [35-37]. In this study, the synthesized compounds were analyzed against α-amylase and α-glucosidase enzymes. The structure of these enzymes was retrieved from the protein data bank (PDB) with searching codes **1b2y** &**3w37**.

In the first step, the protein was prepared by using DSV by removing water molecules and already present ligands were removed saving both the target protein as well as a prepared ligand in PDB format. The process was further carried out in an auto dock in which polar hydrogen and Kollman and gasteiger charges were added to the protein. The selected ligand was also prepared by using a torsion tree to detect the root. Moreover, the configuration file was generated along with the X, Y, and Z axis saving both ligand and protein in PDBQT format in the same docking folder. At the end command prompt was used to generate varied poses of ligand thus, 9 different poses were obtained in PDBQT format. The dock protein and ligand were then opened in DSV to identify the binding interaction of the ligand with active sites of the enzyme.


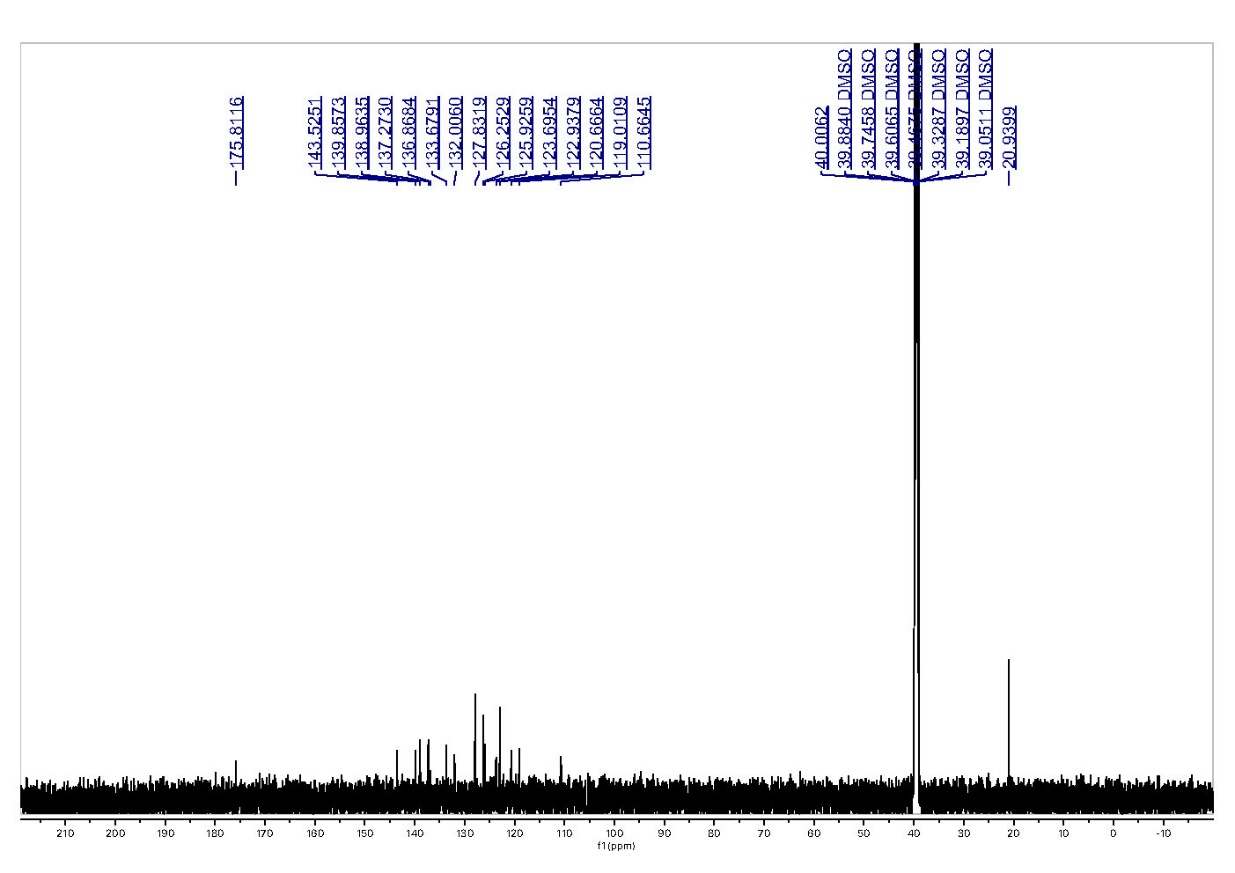


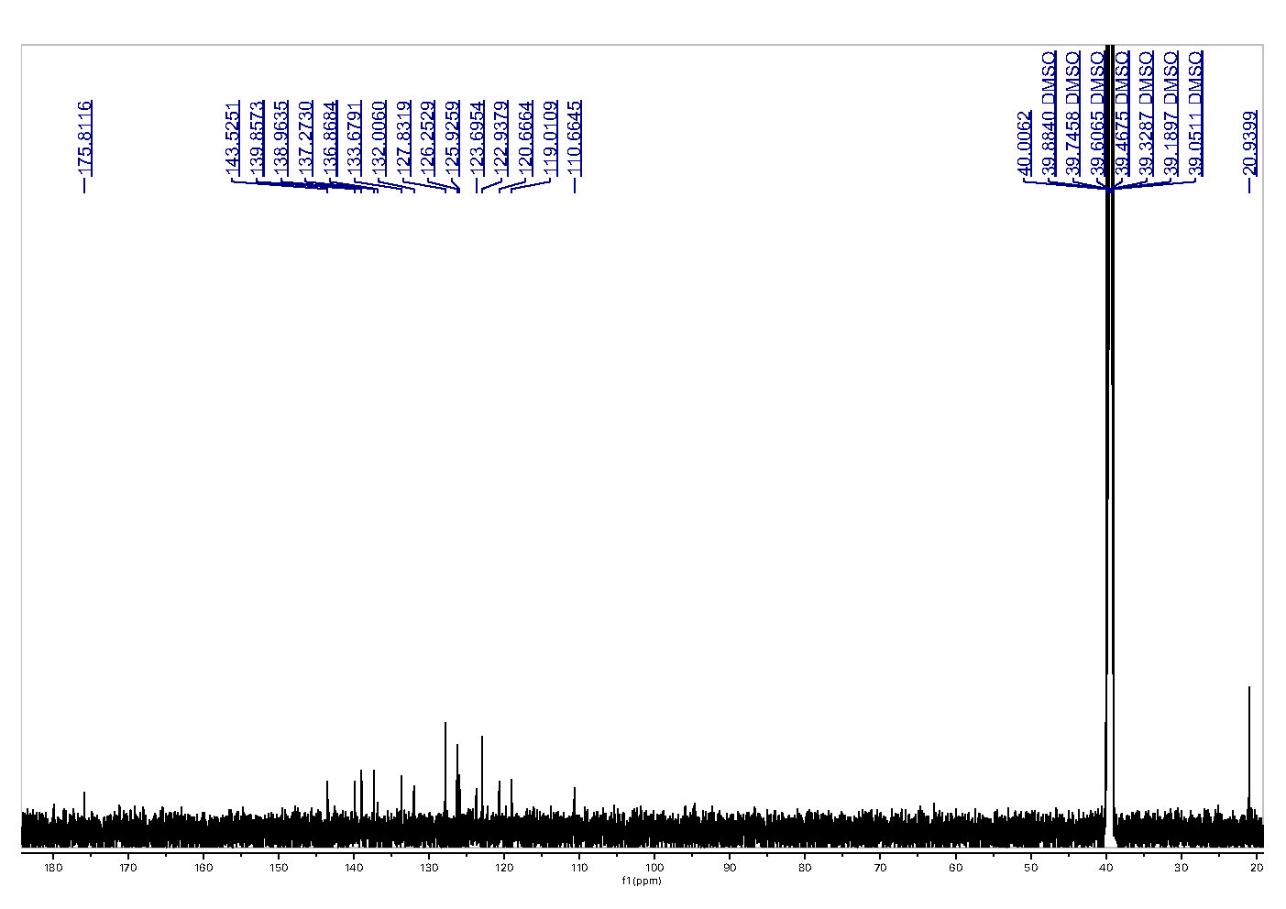


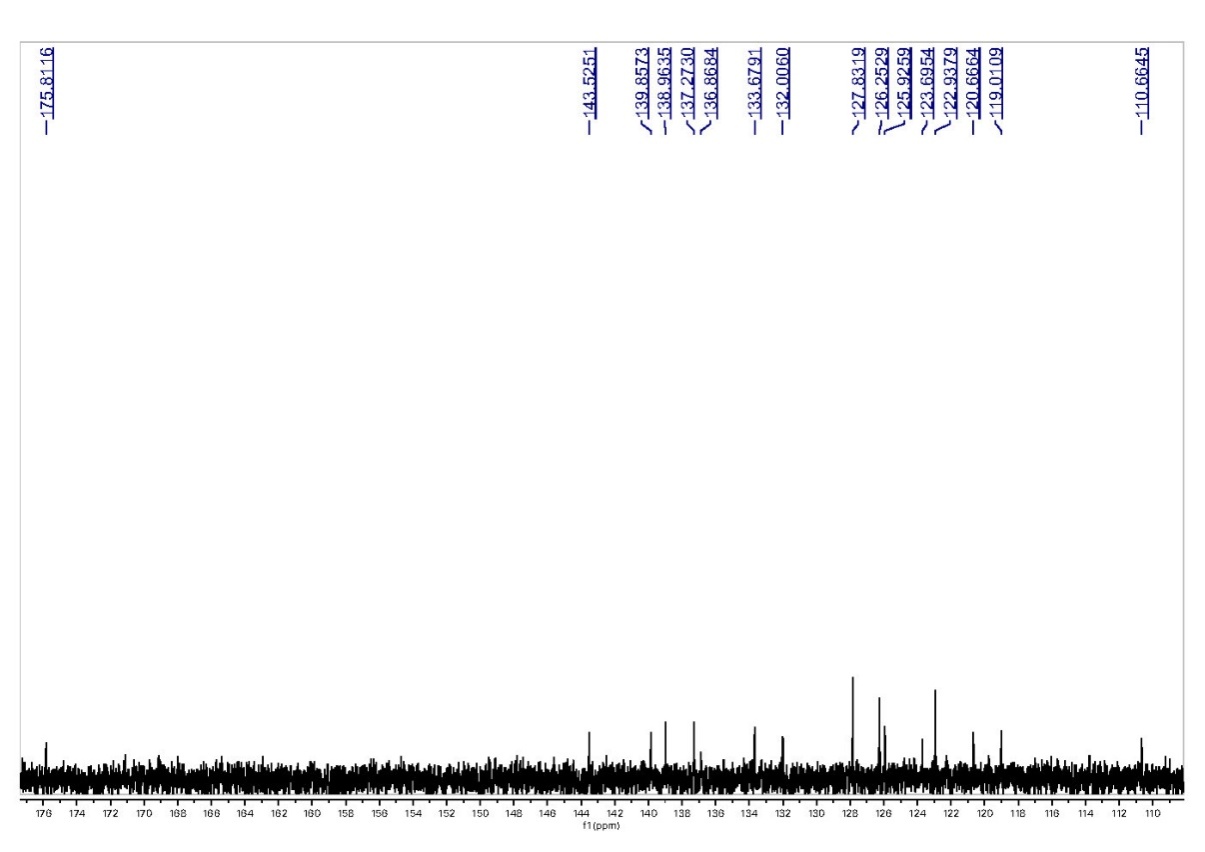


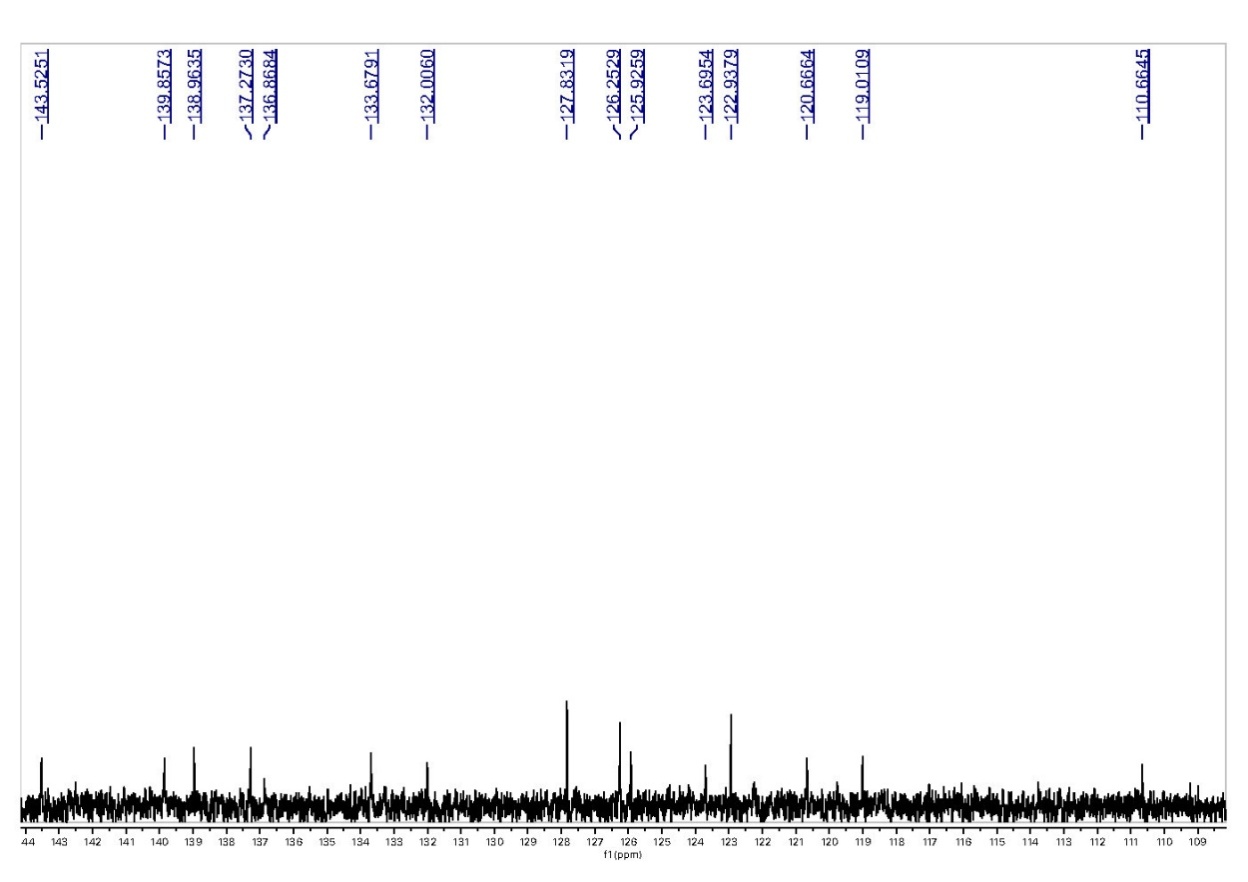


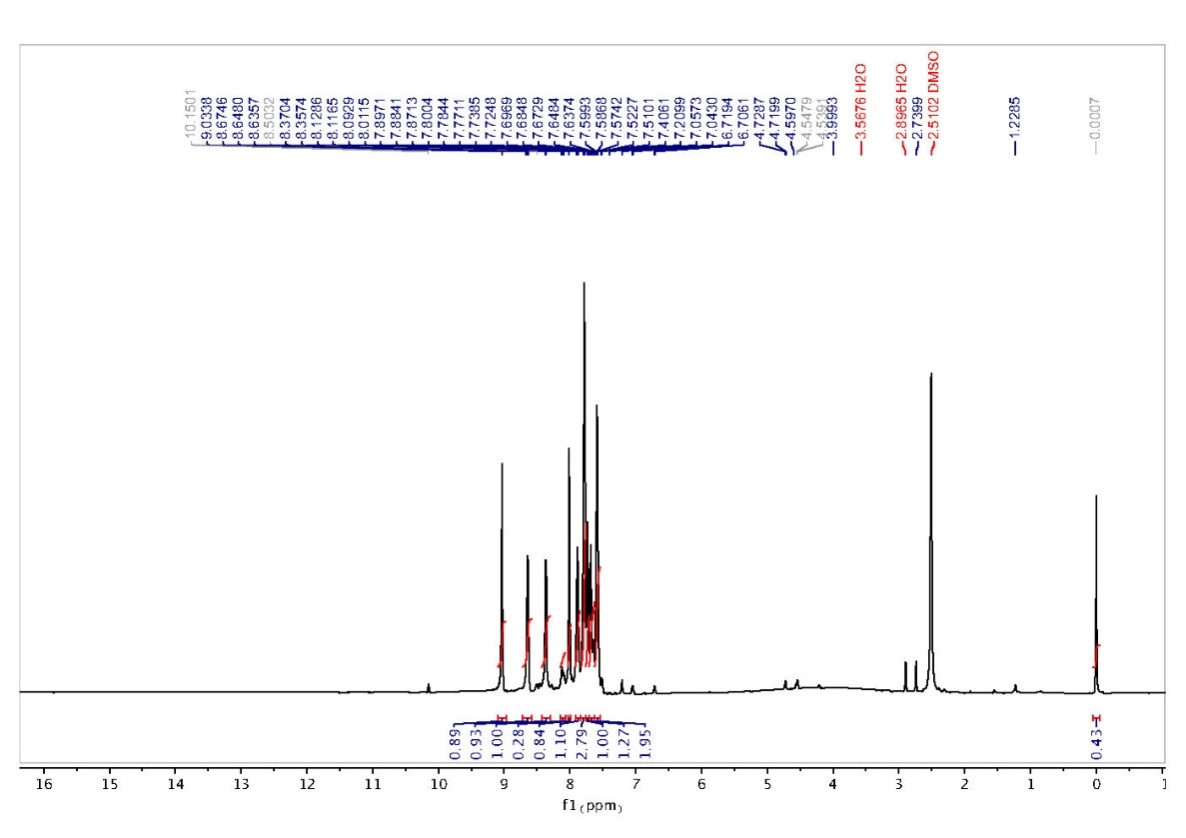


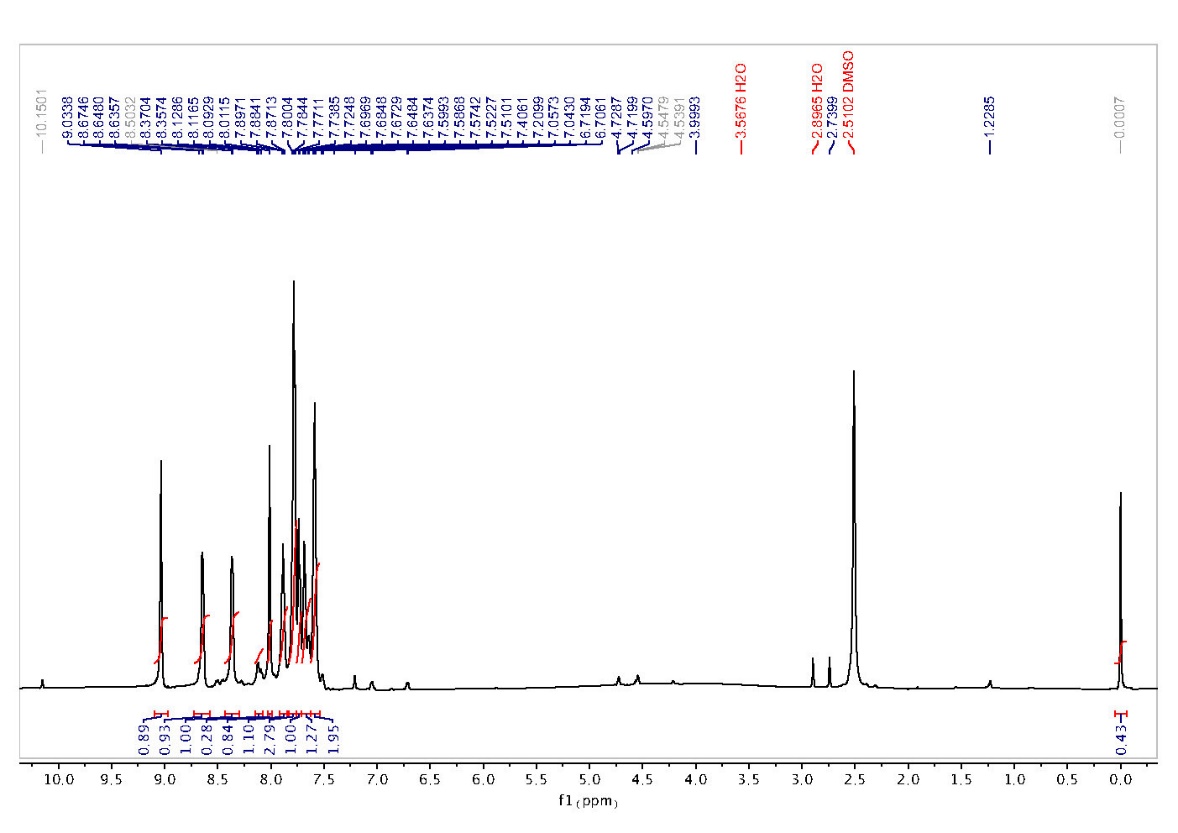


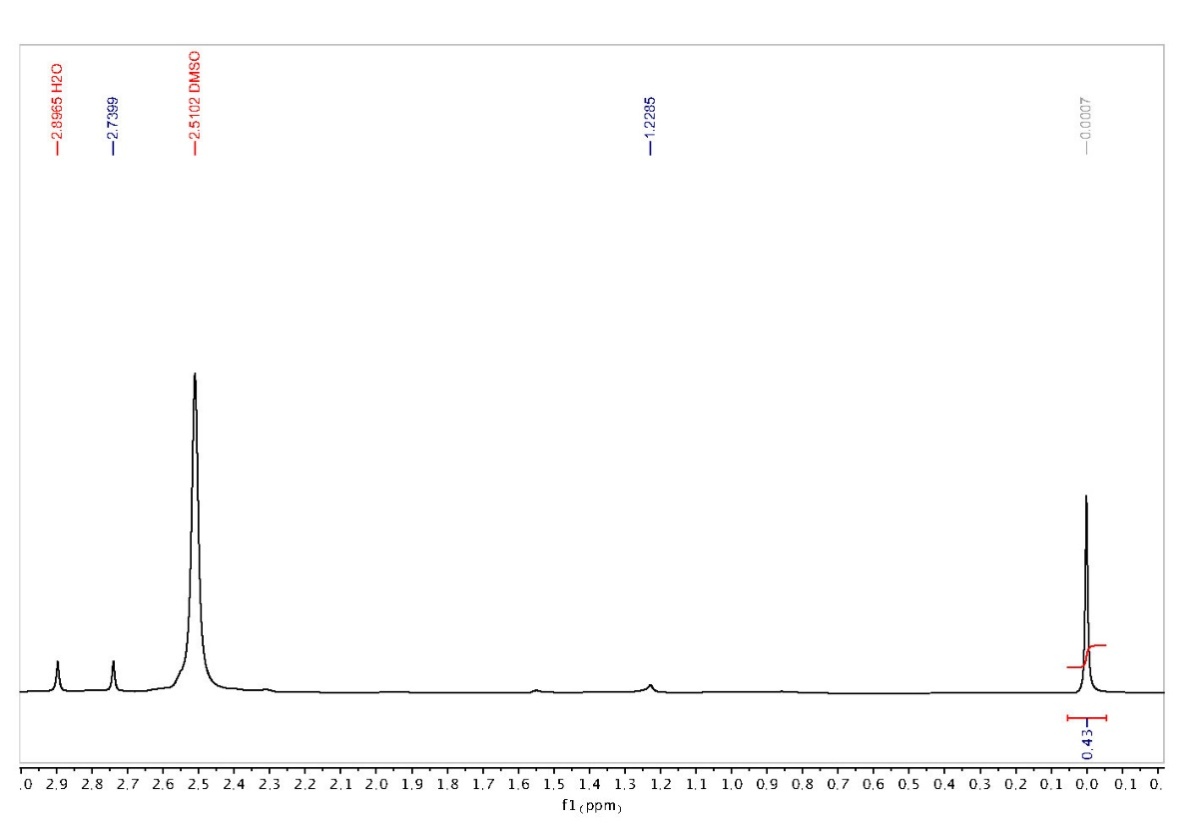


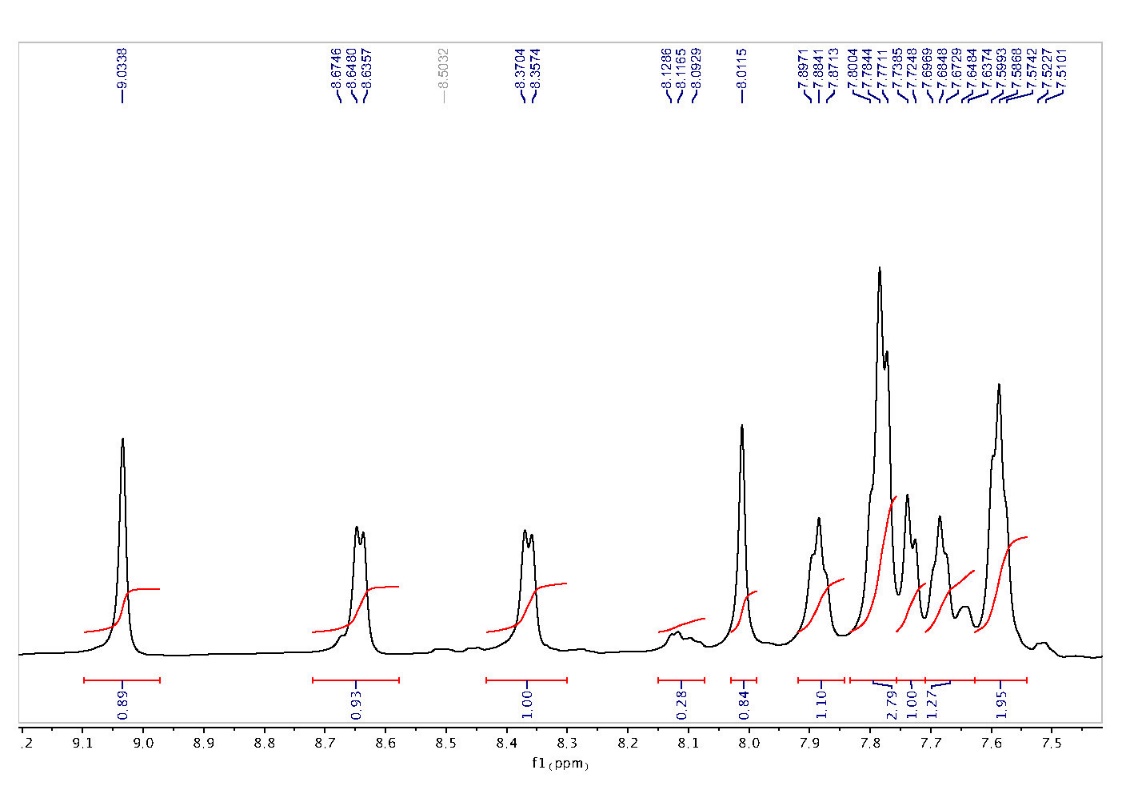


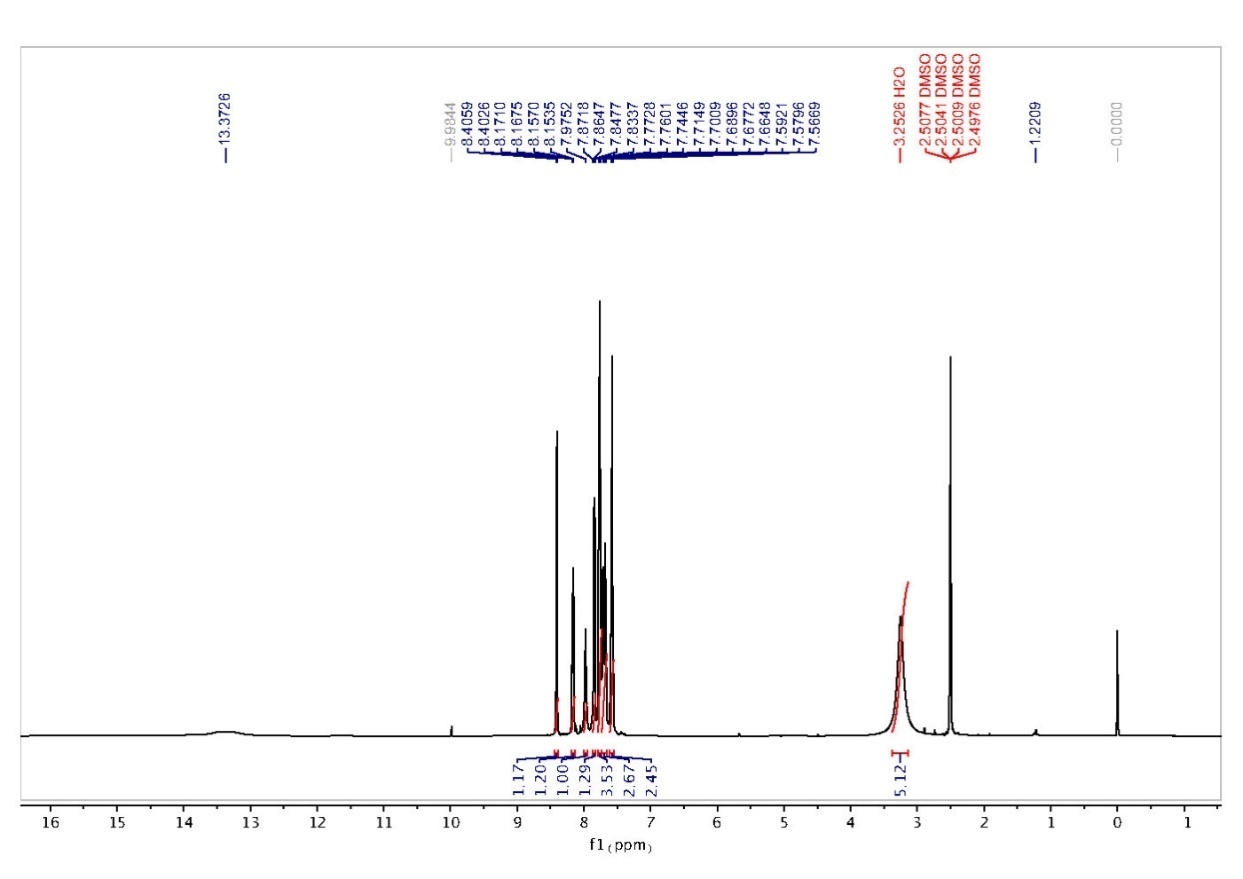


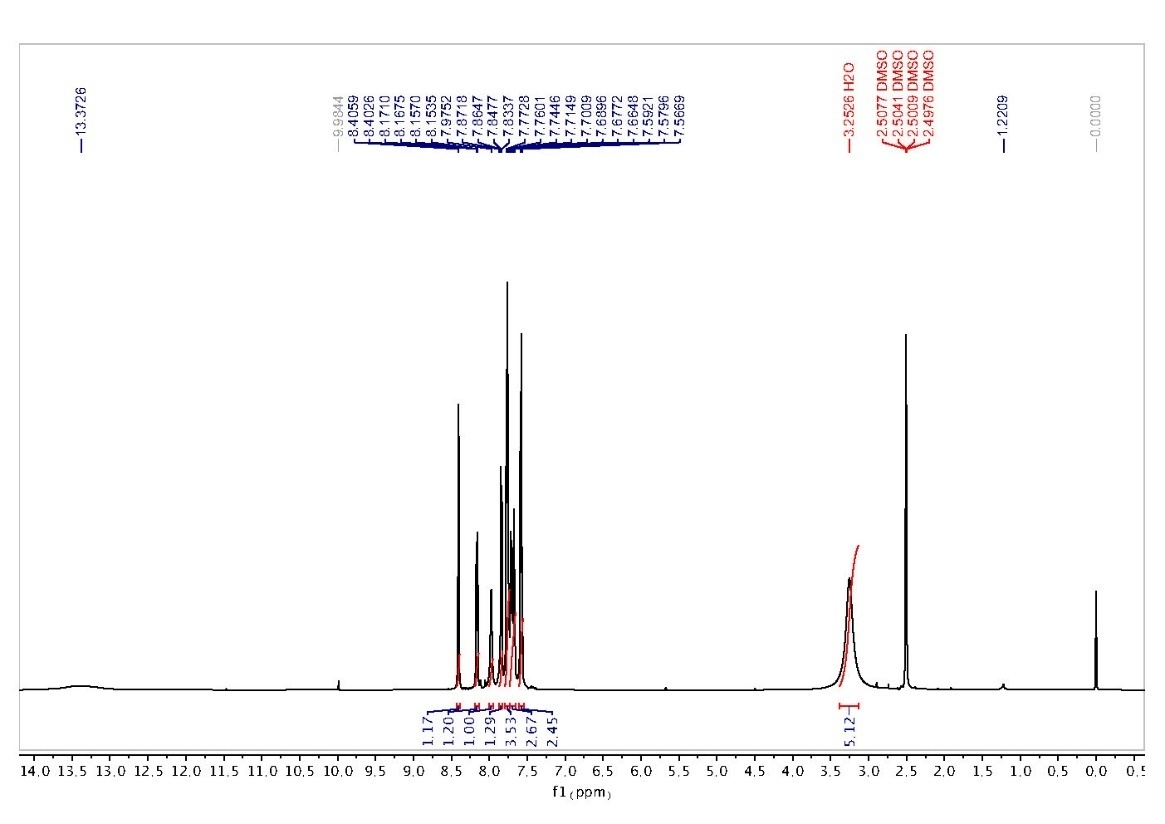


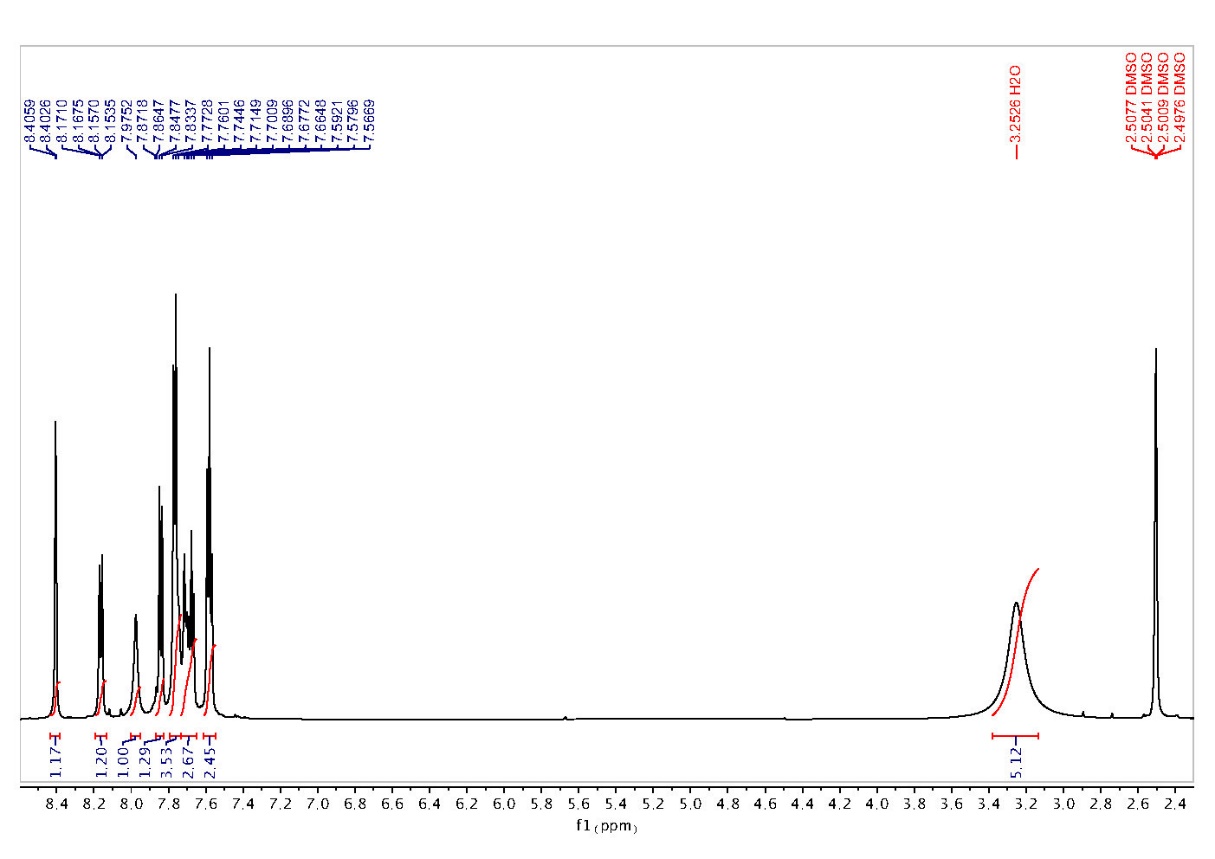


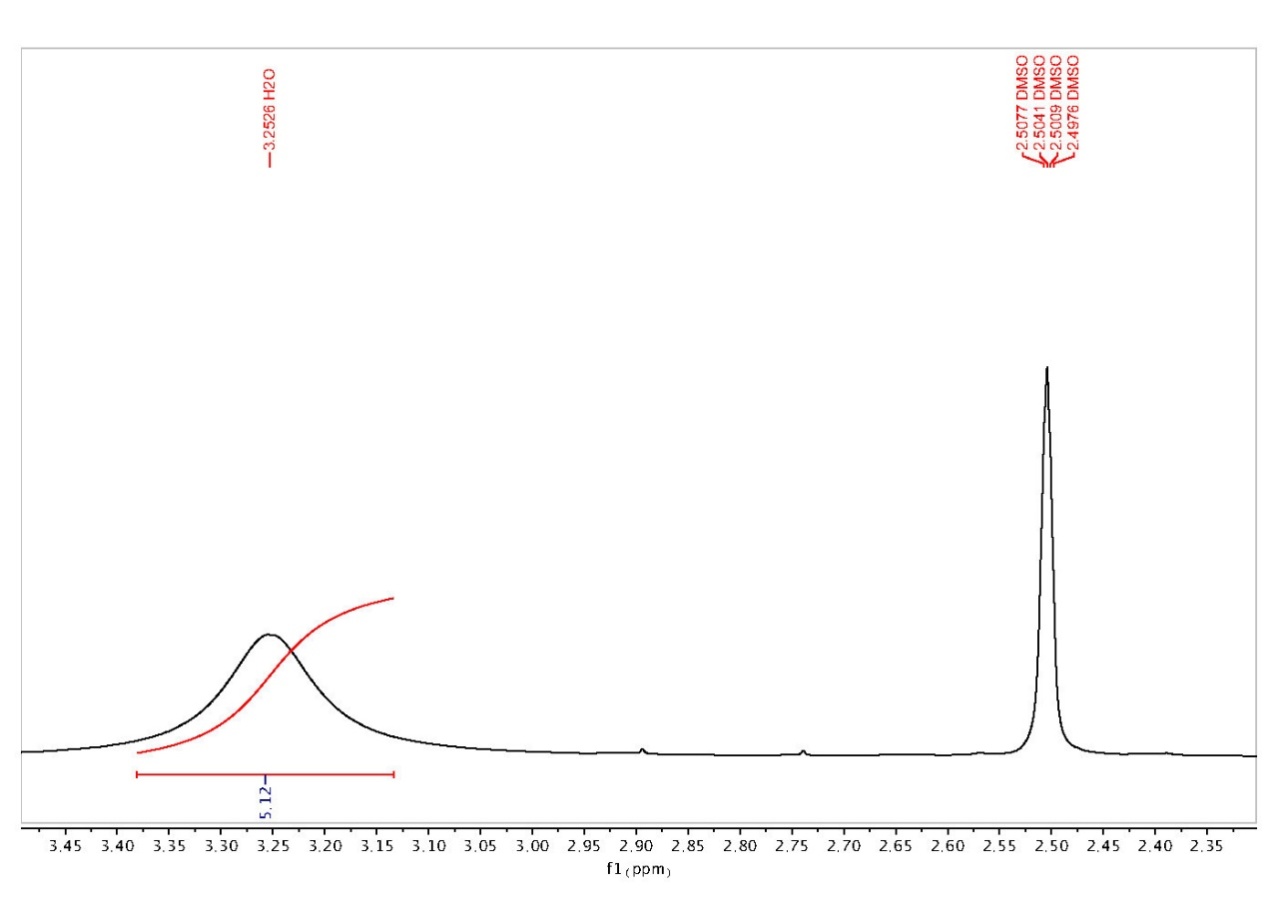


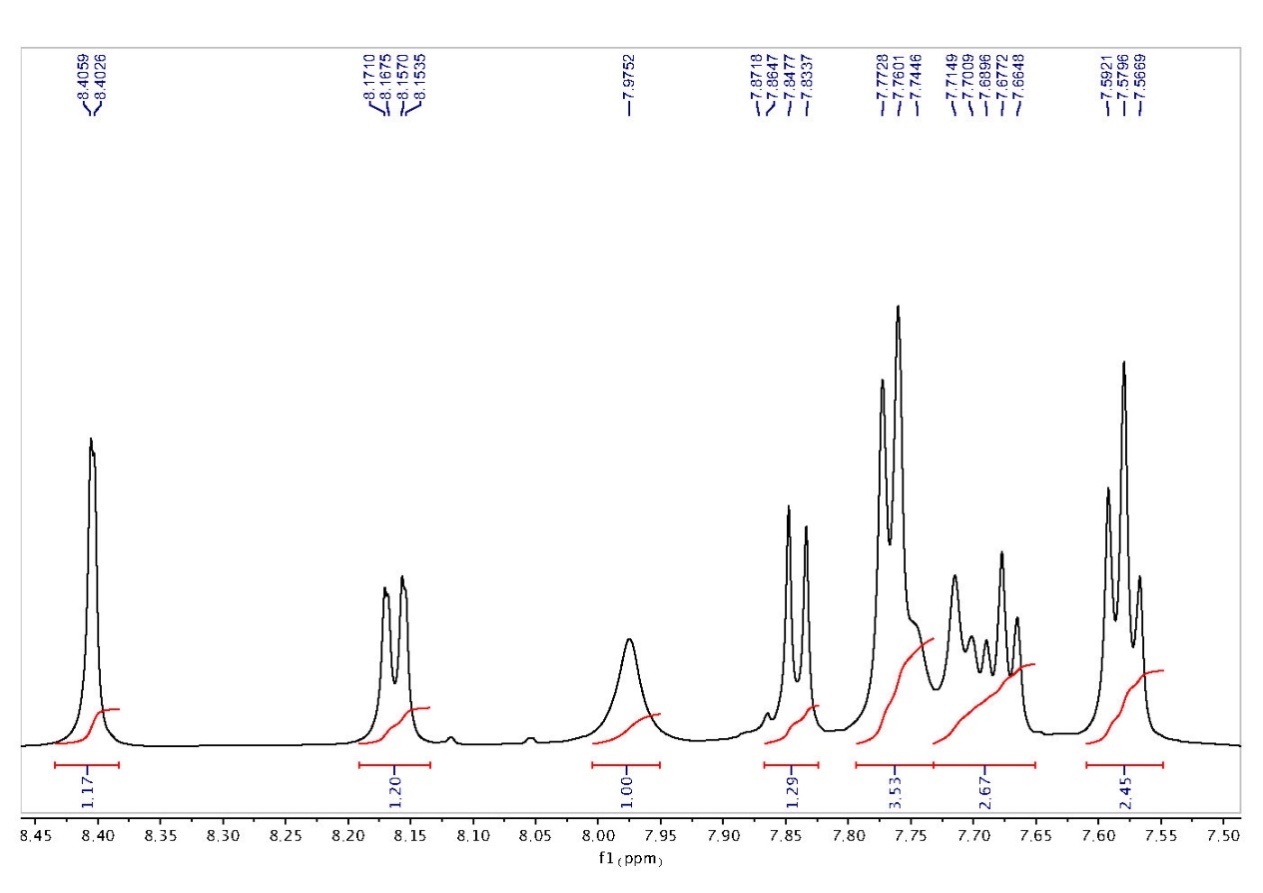


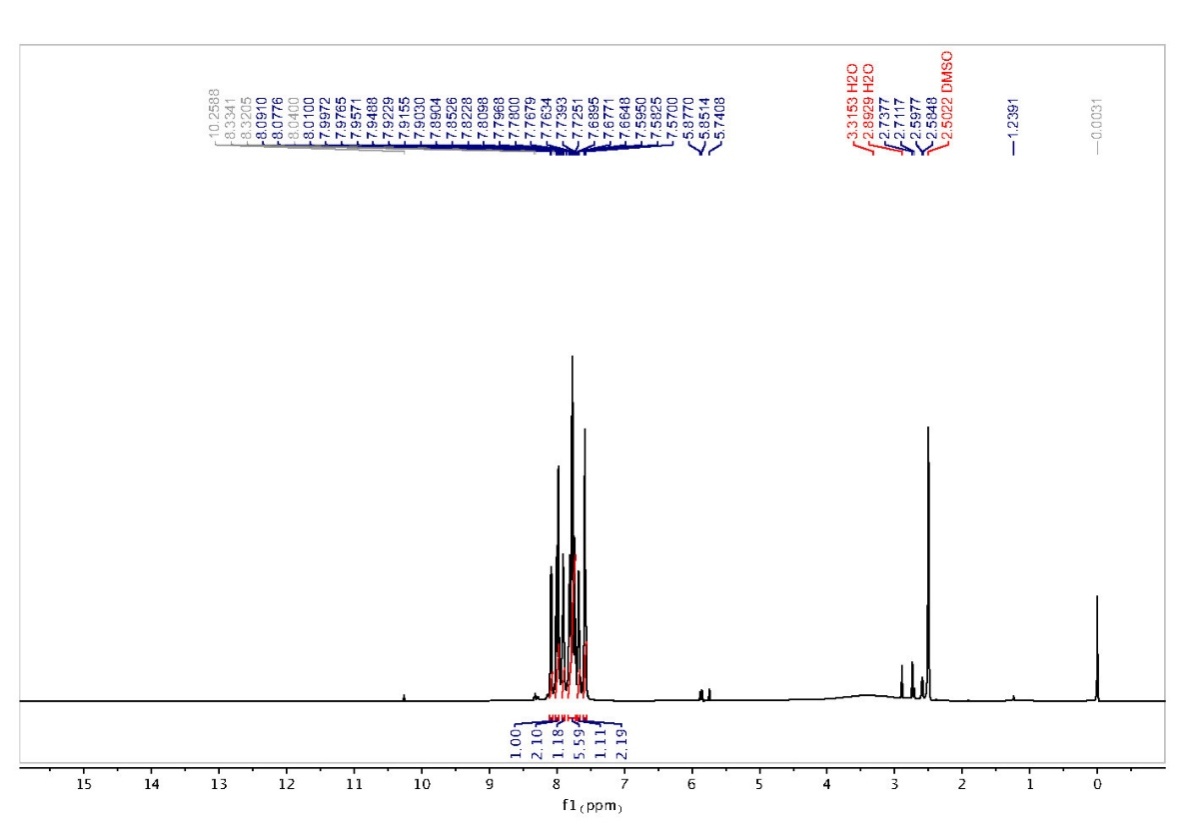


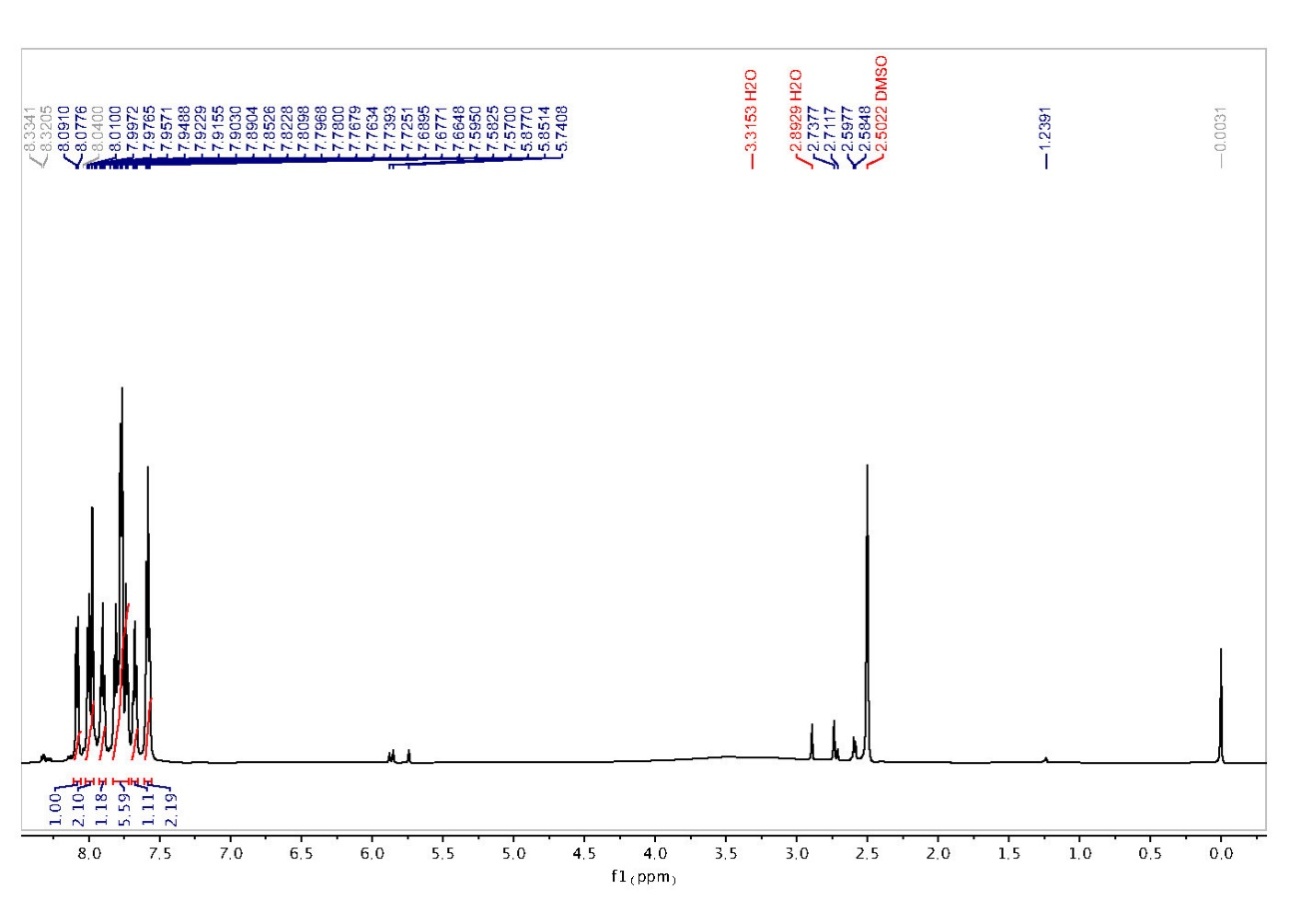


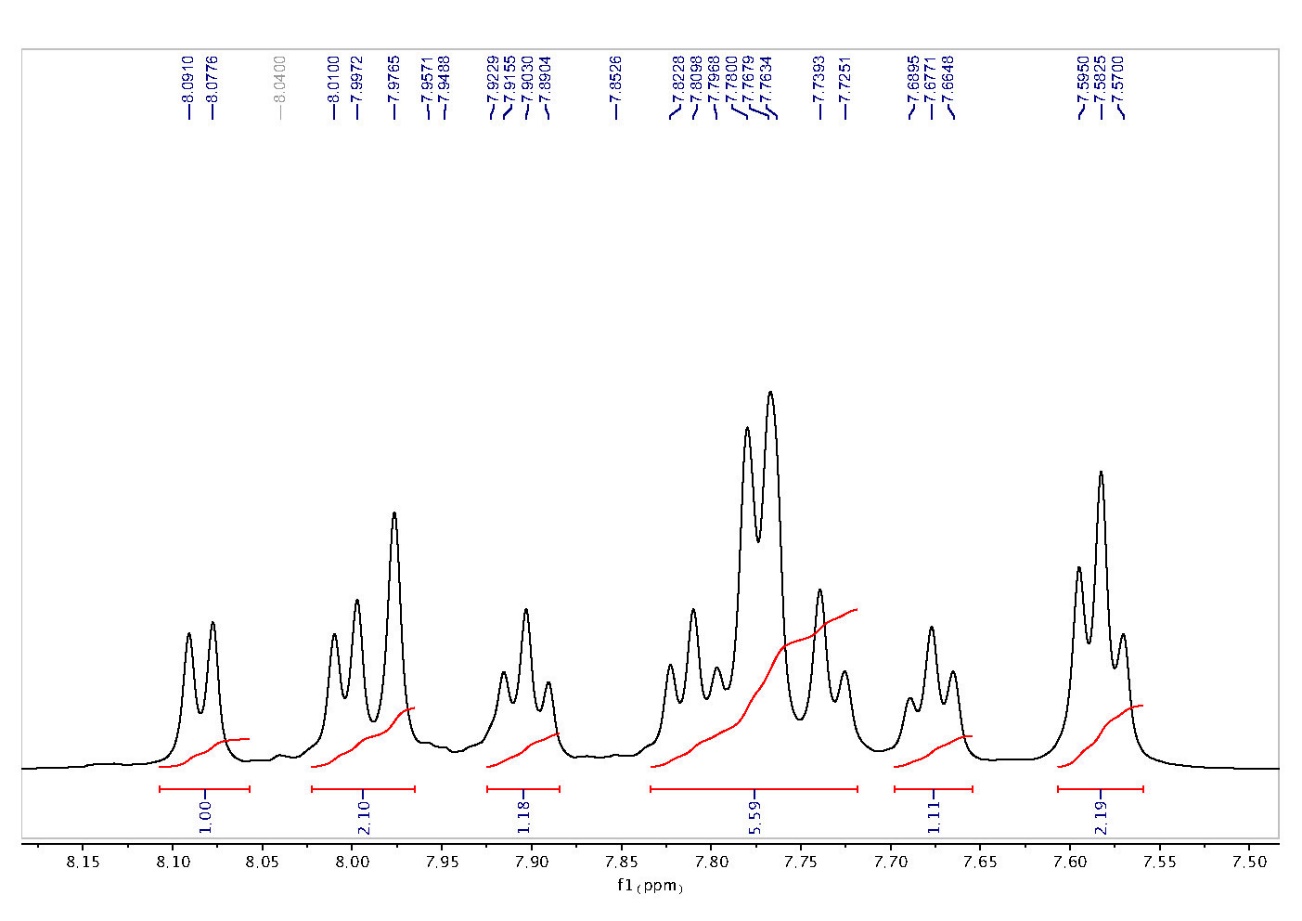


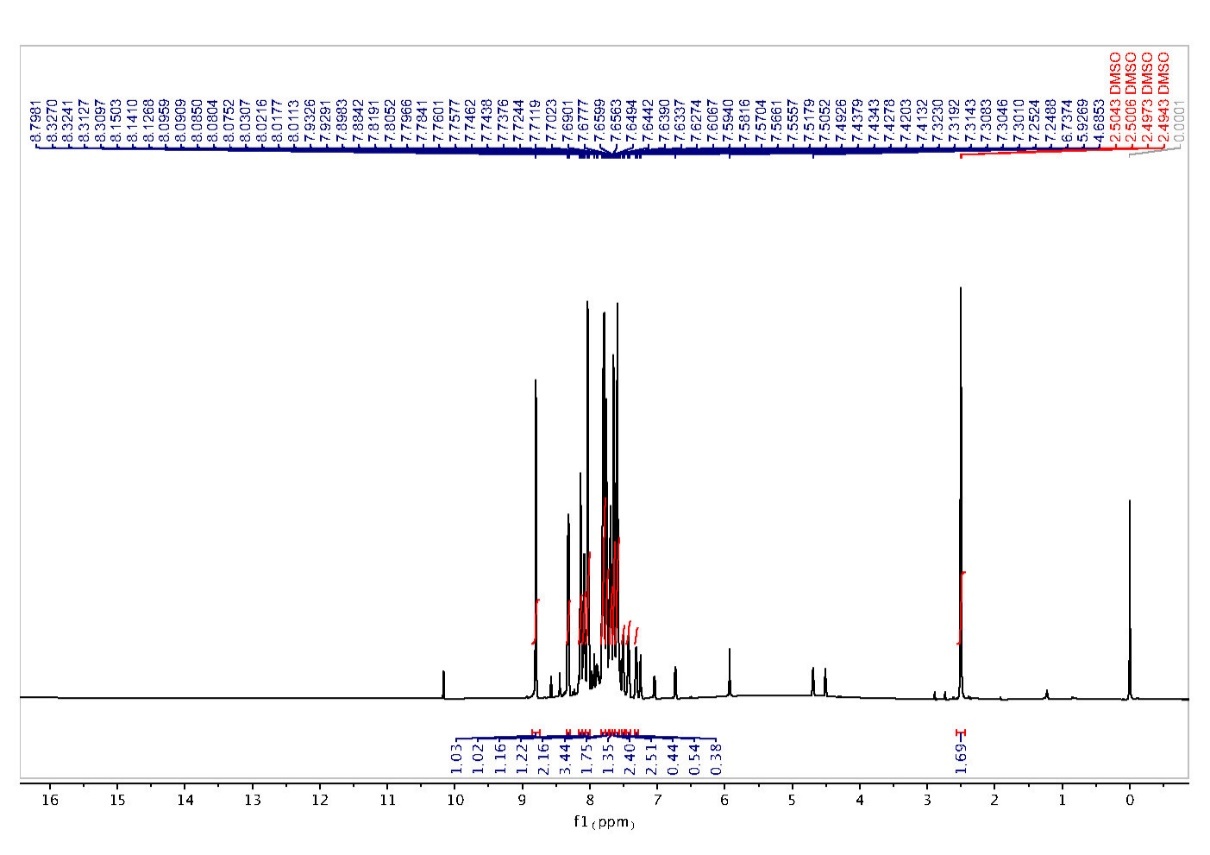


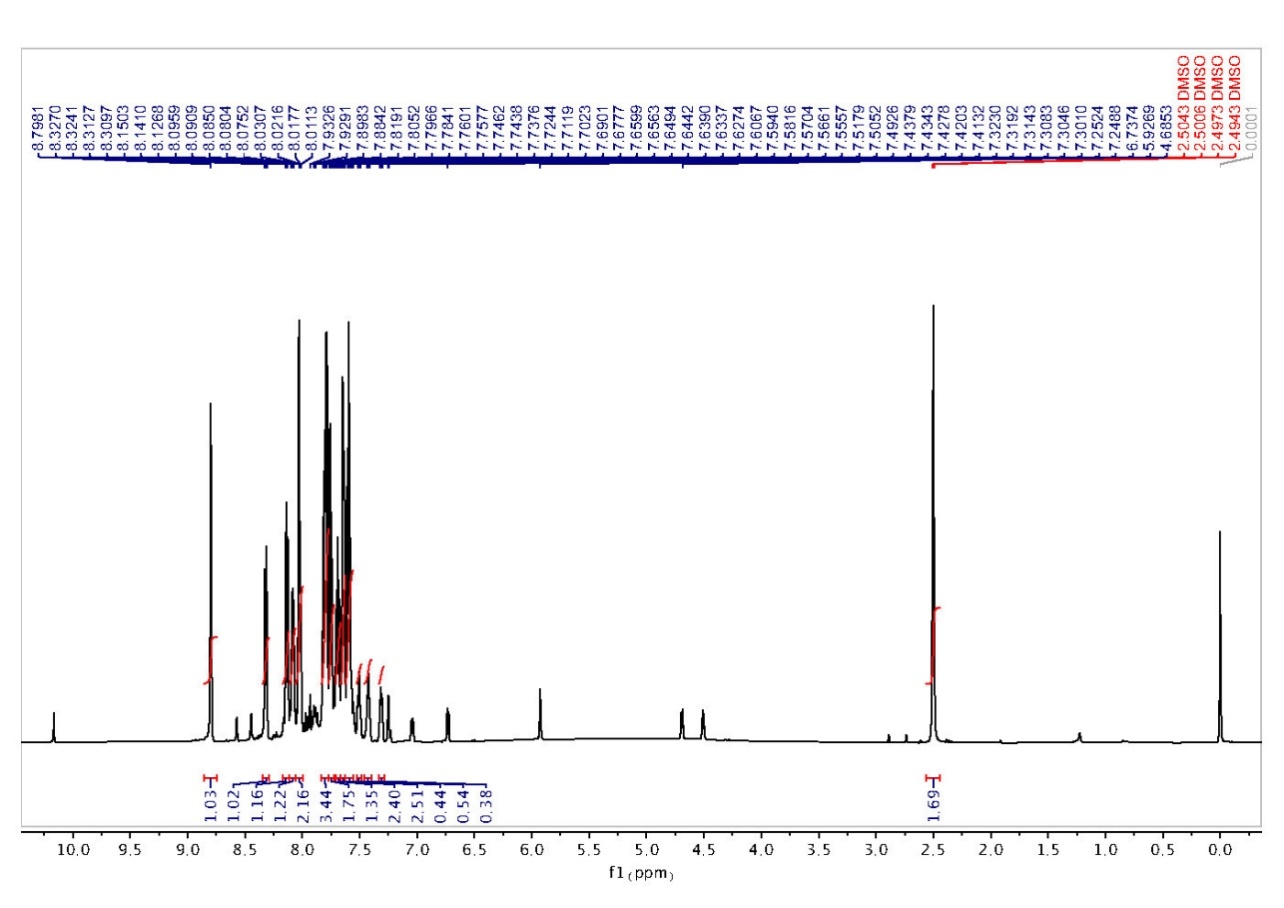


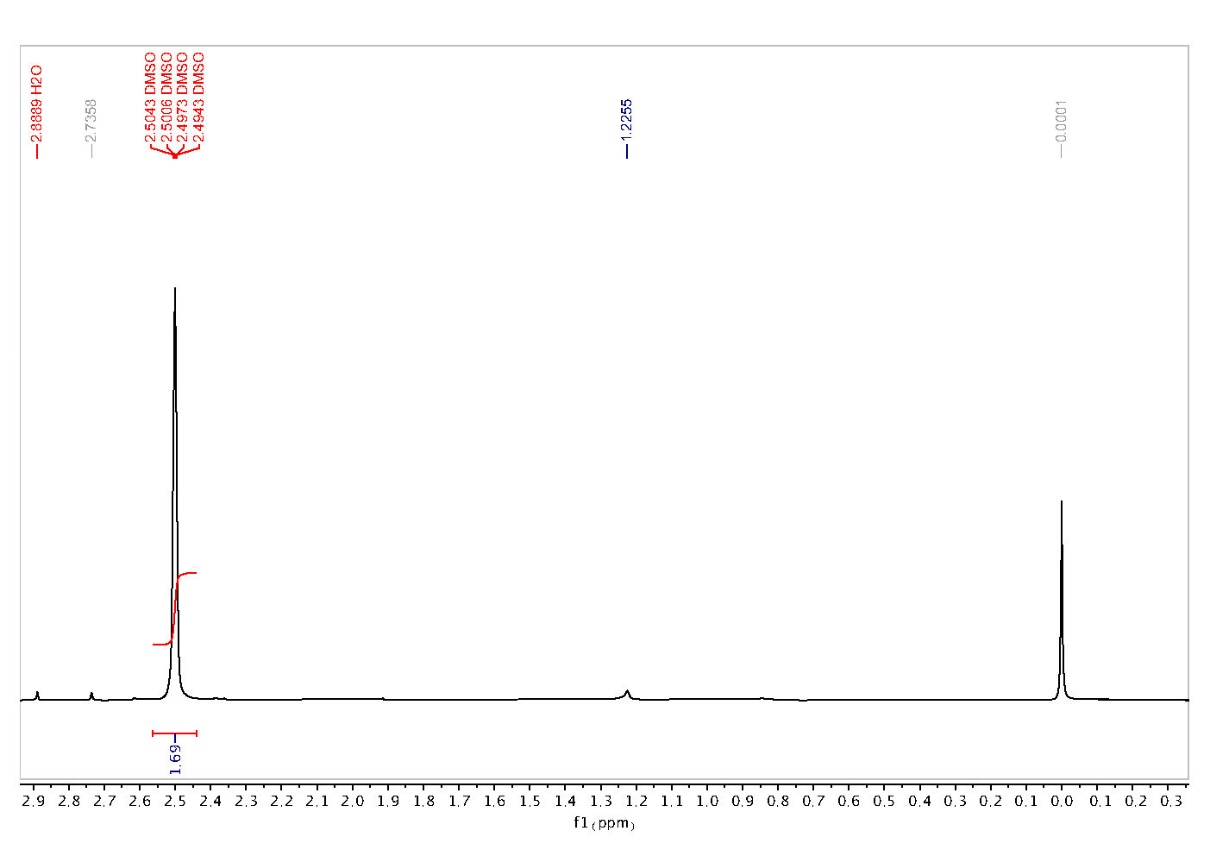


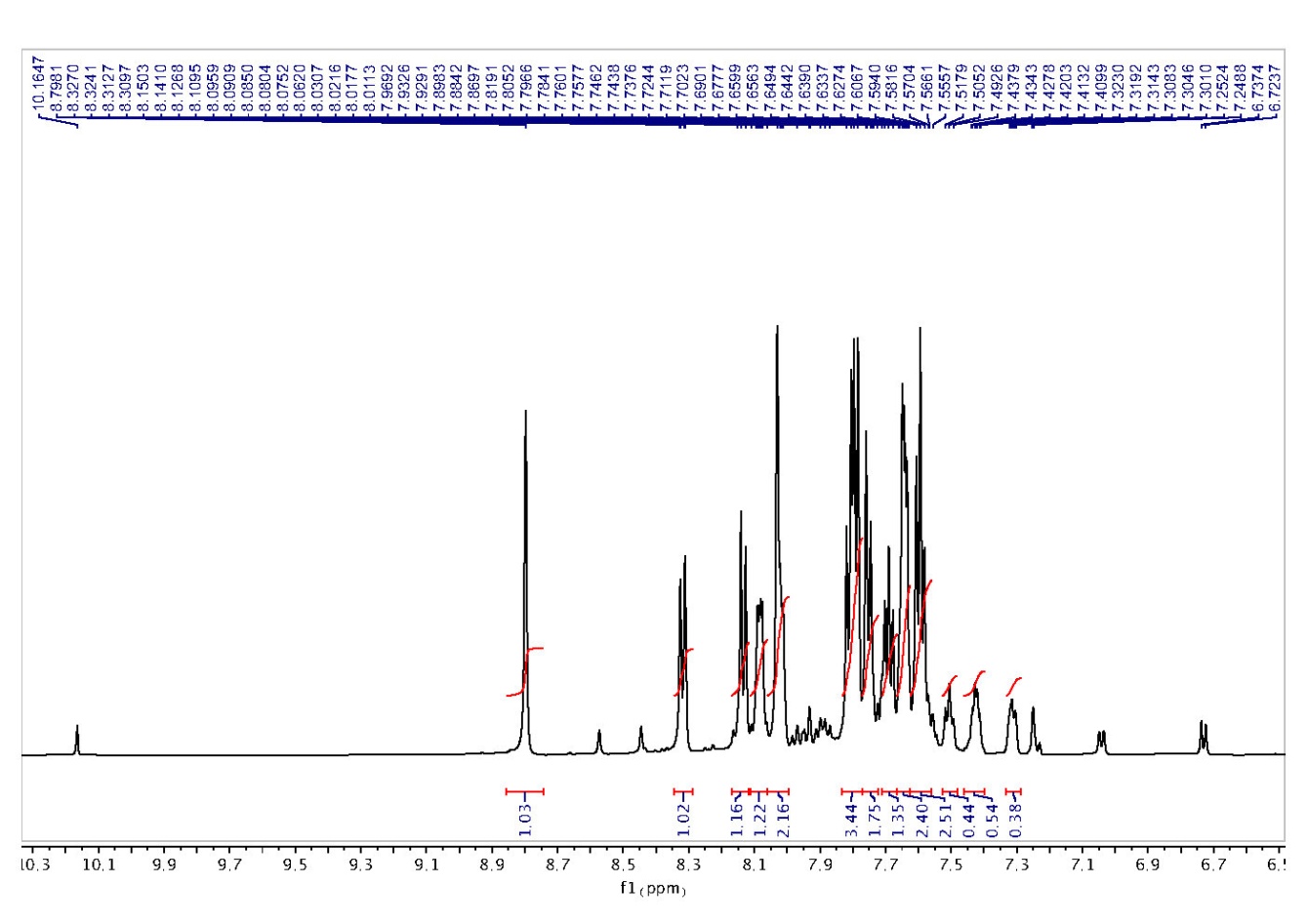


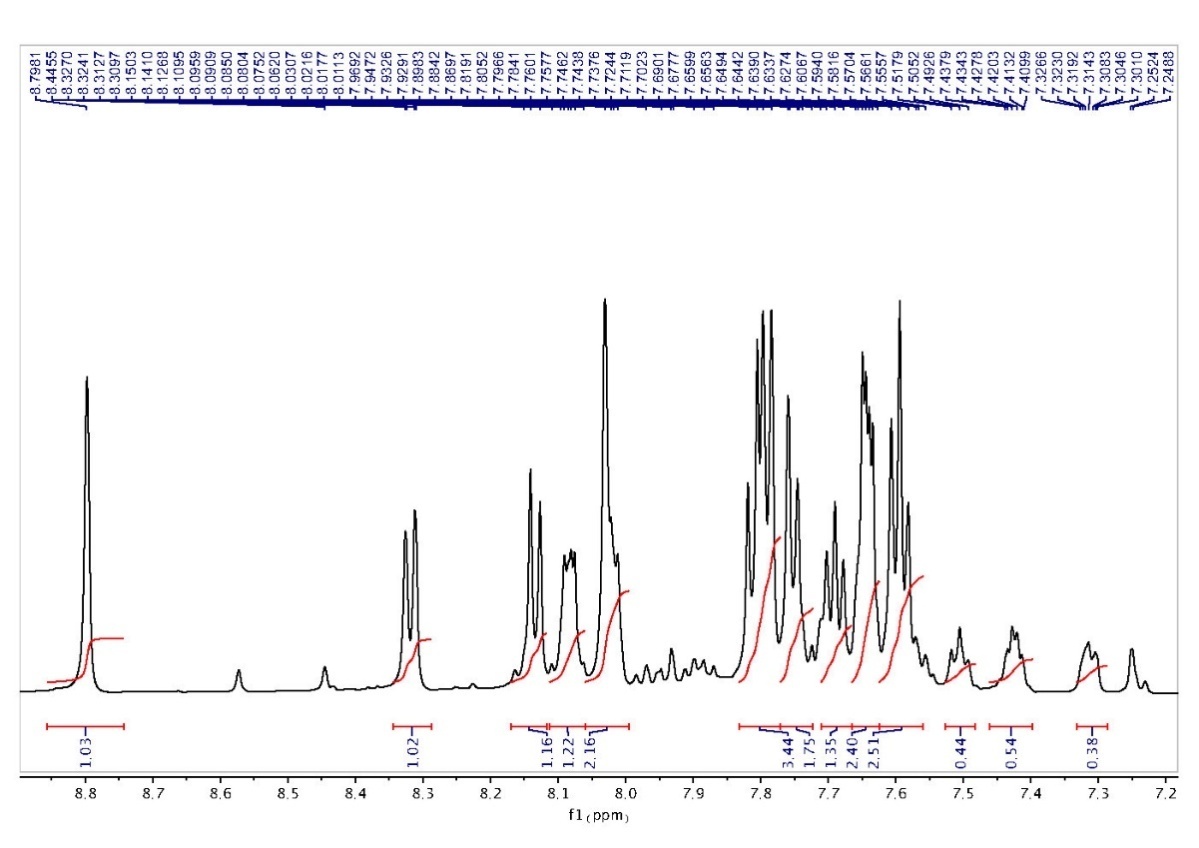


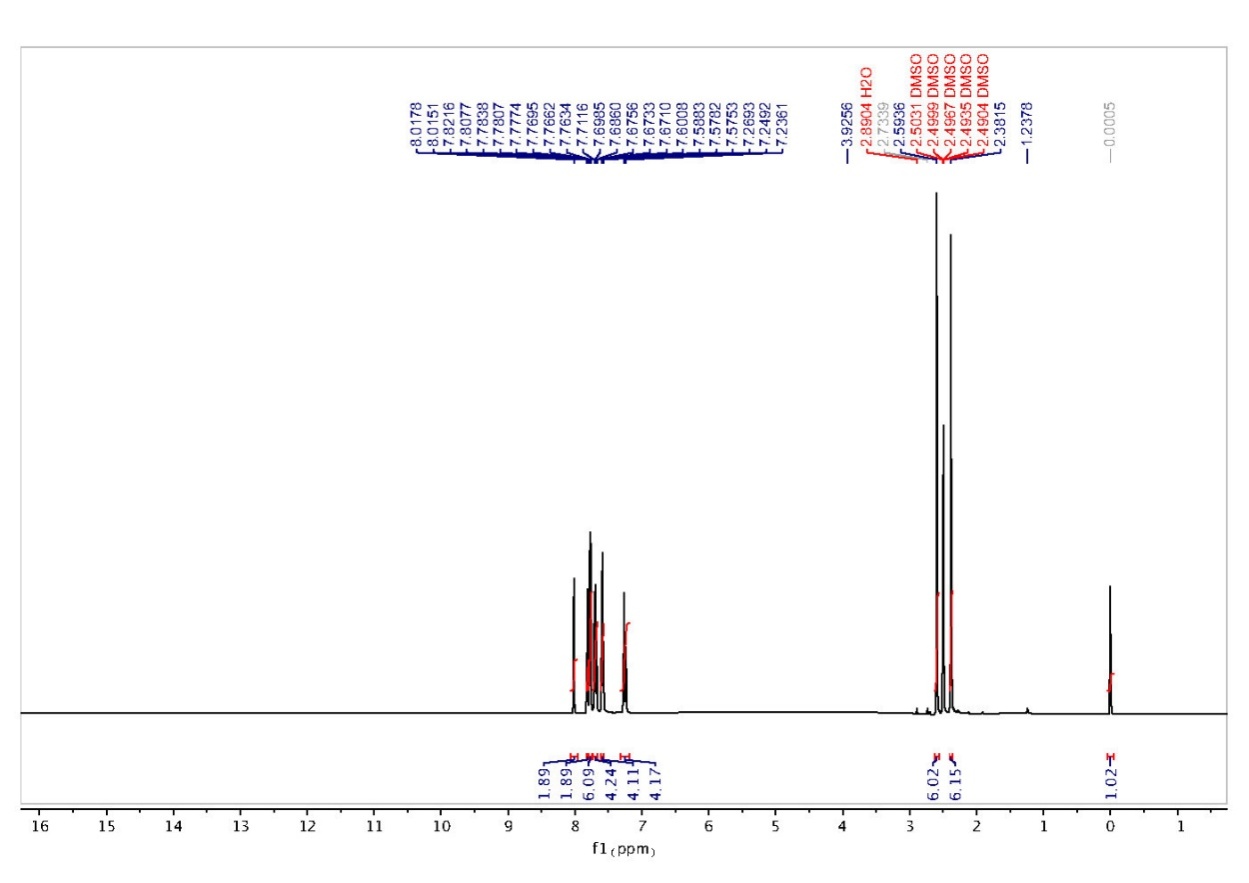


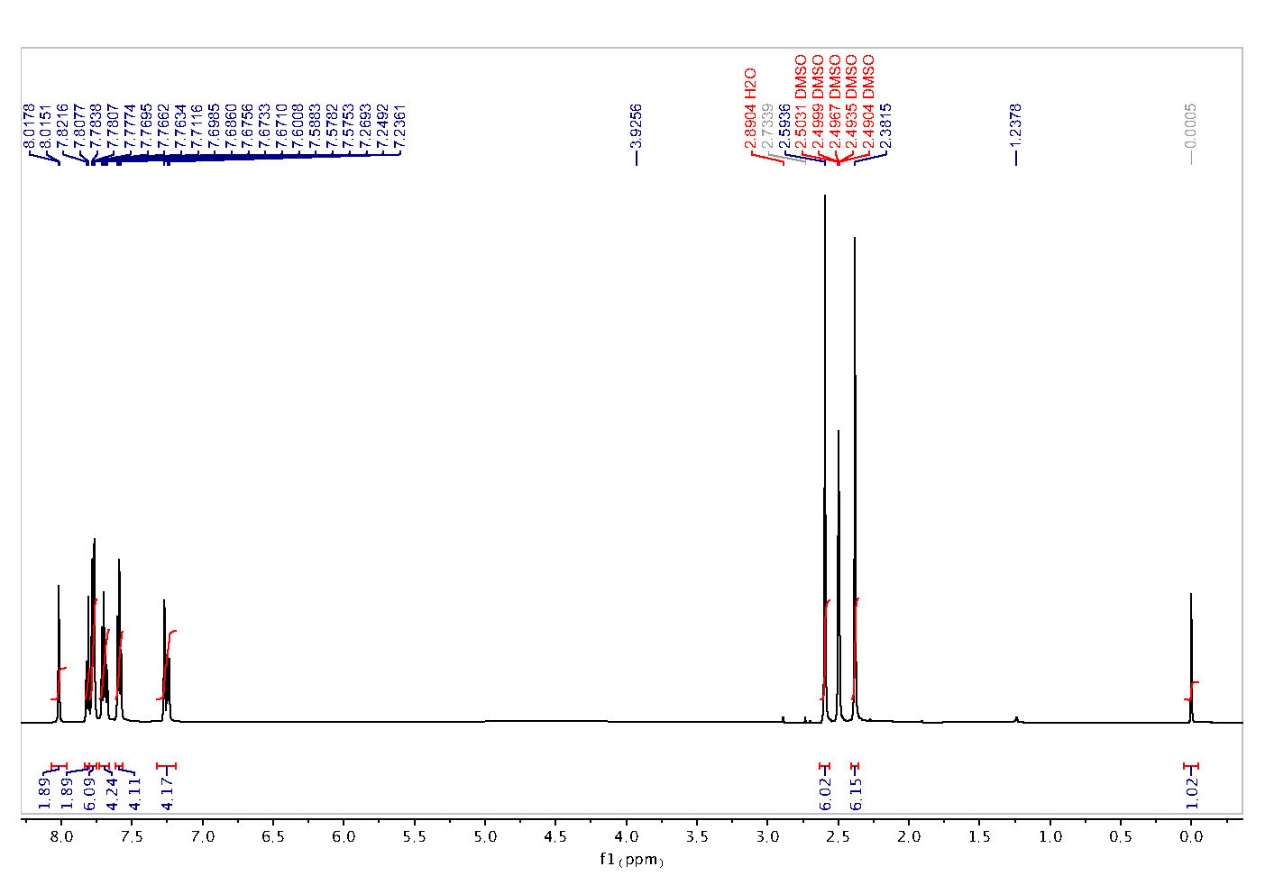


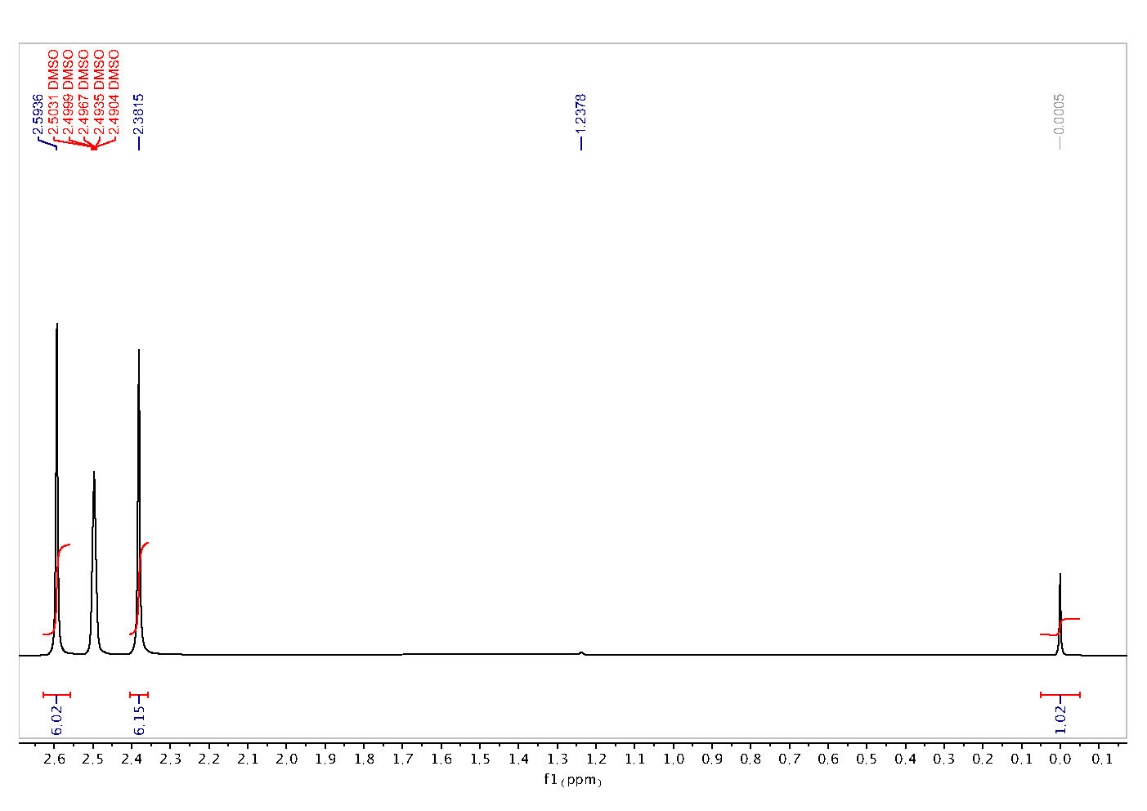


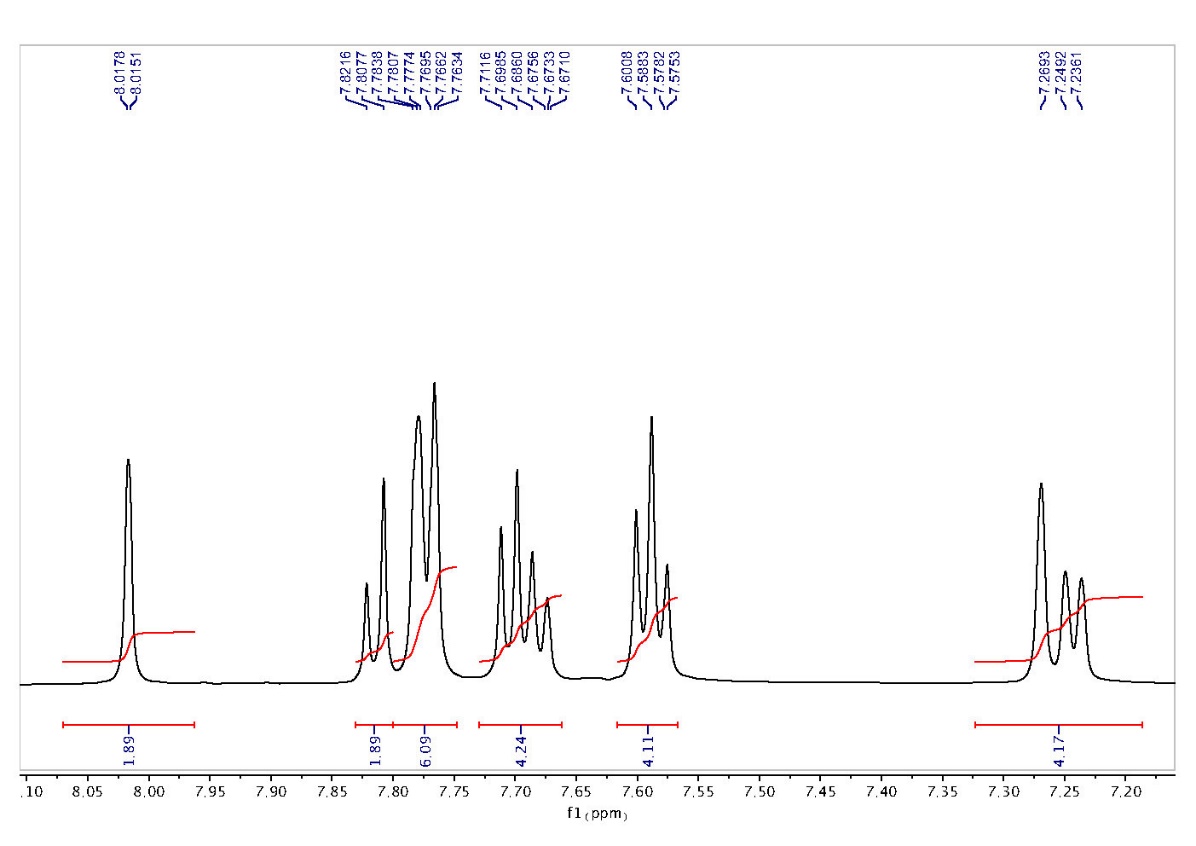


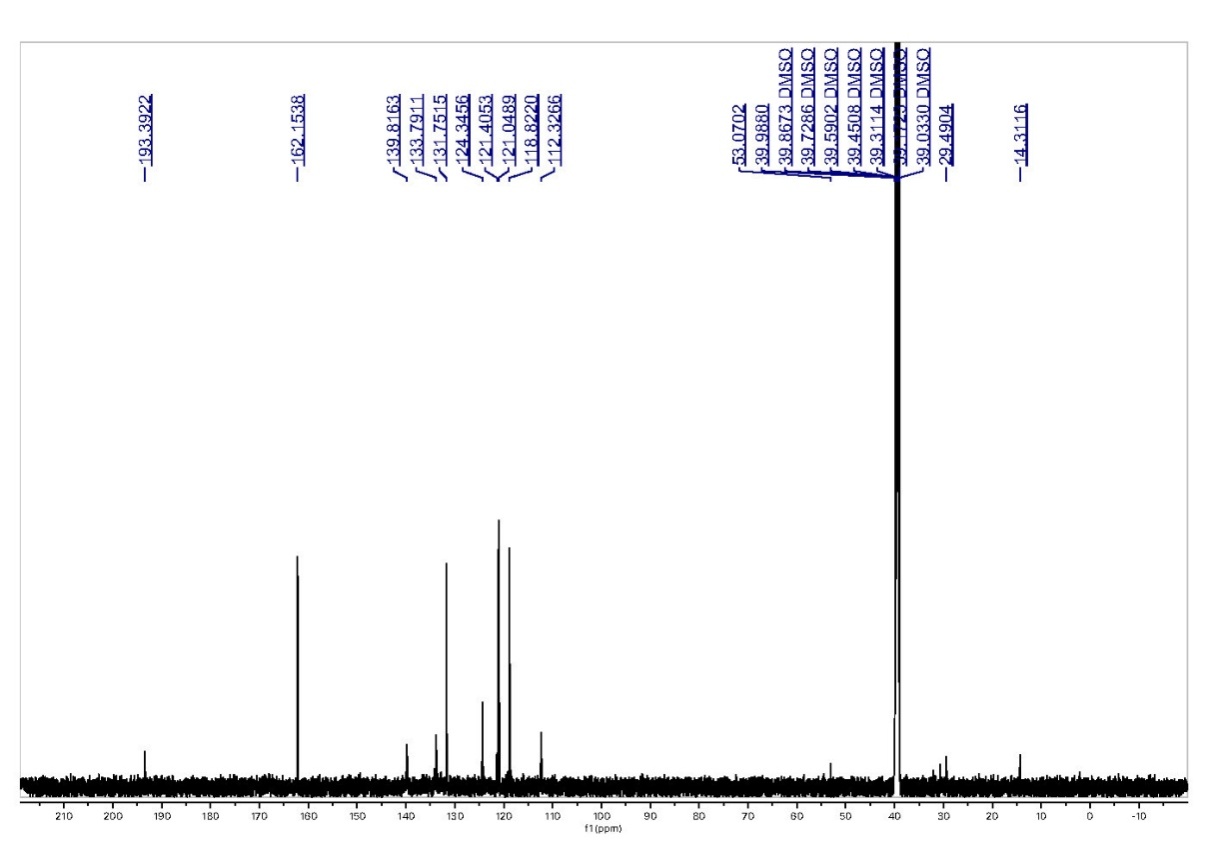


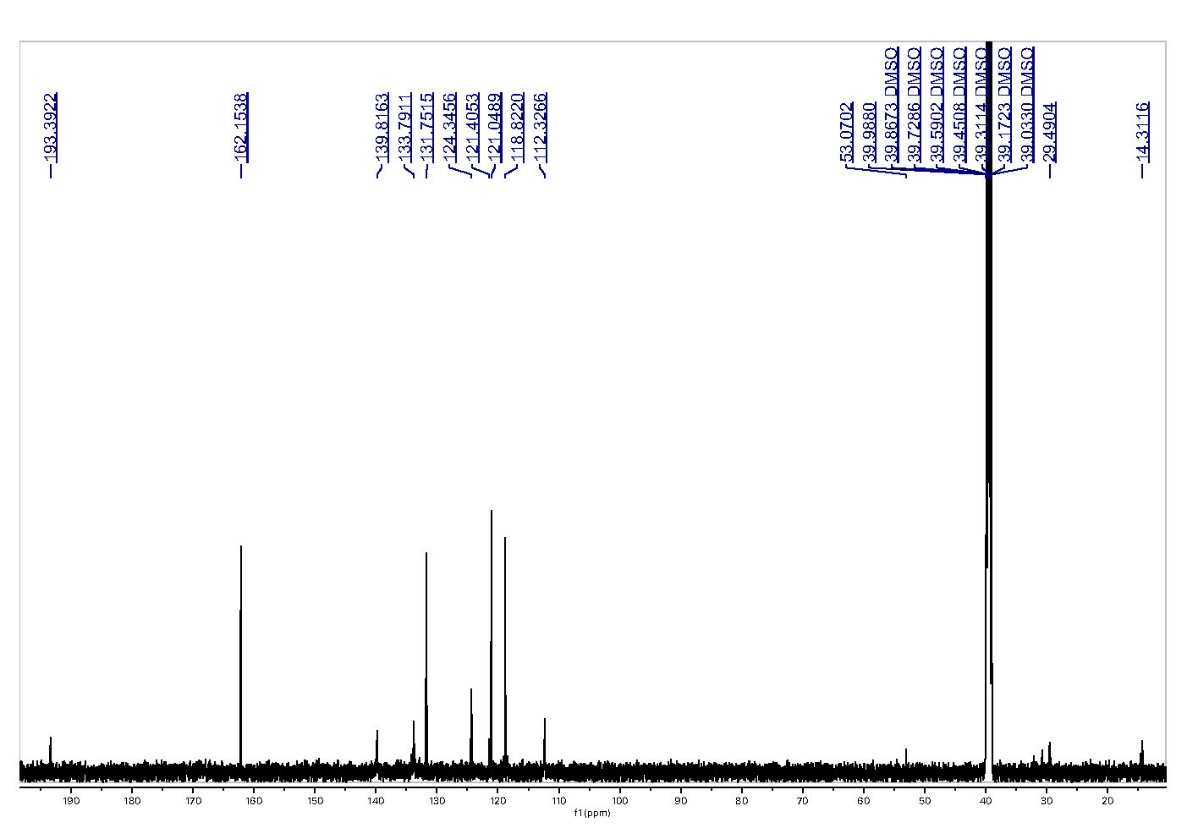


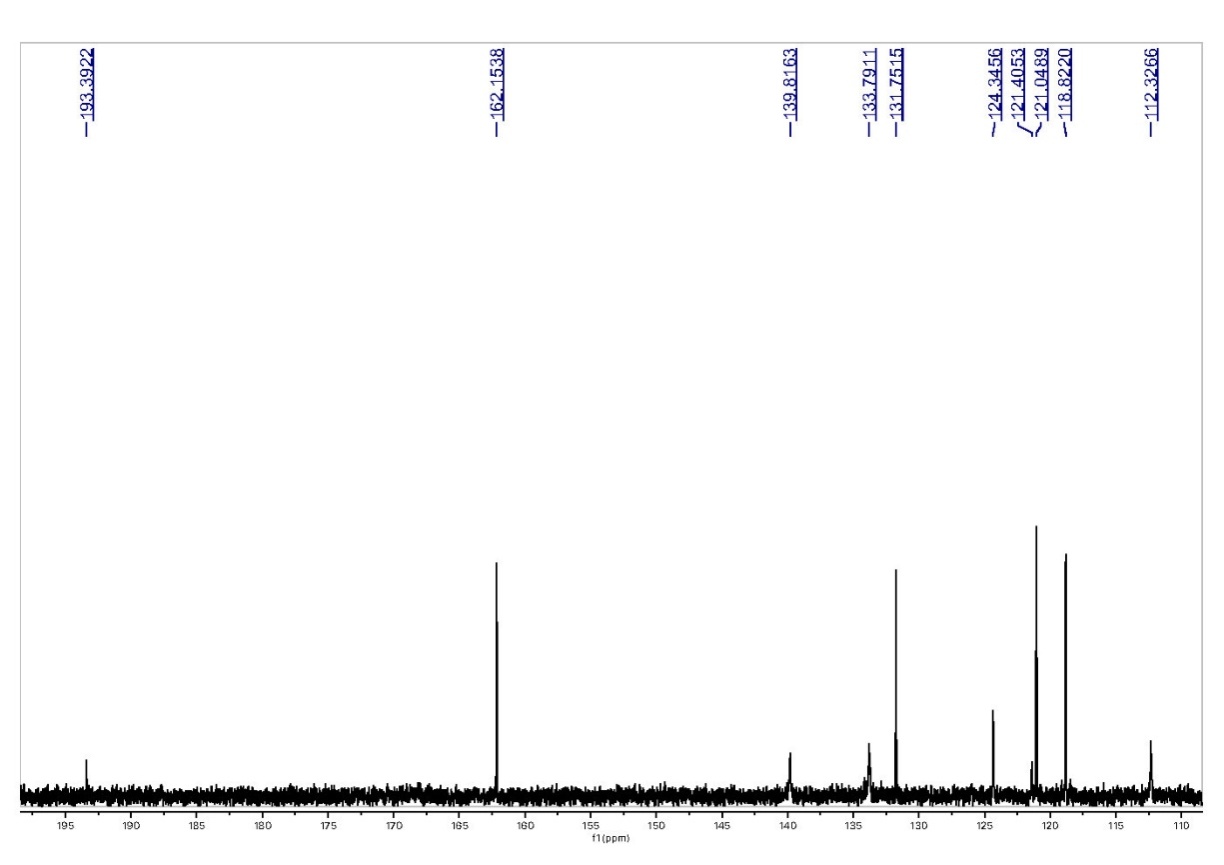


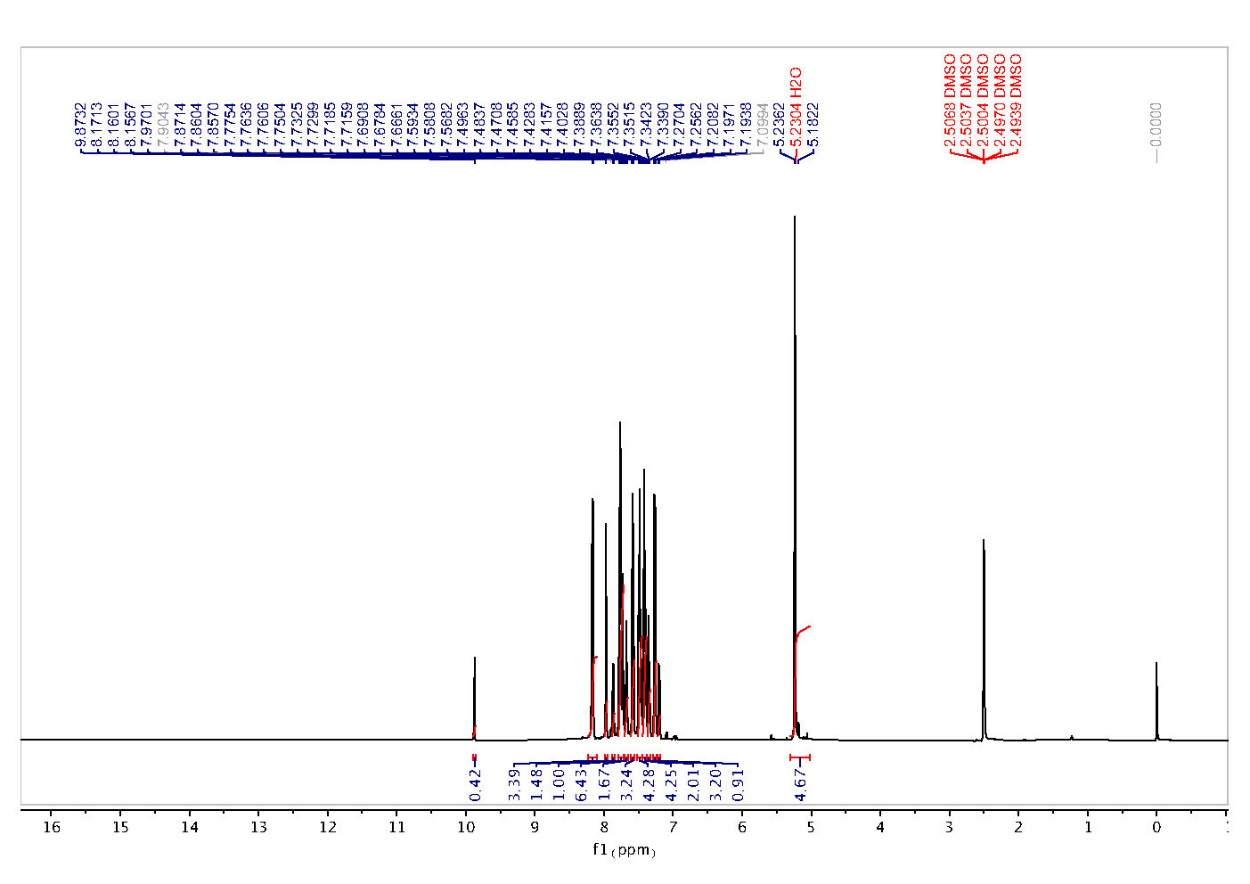


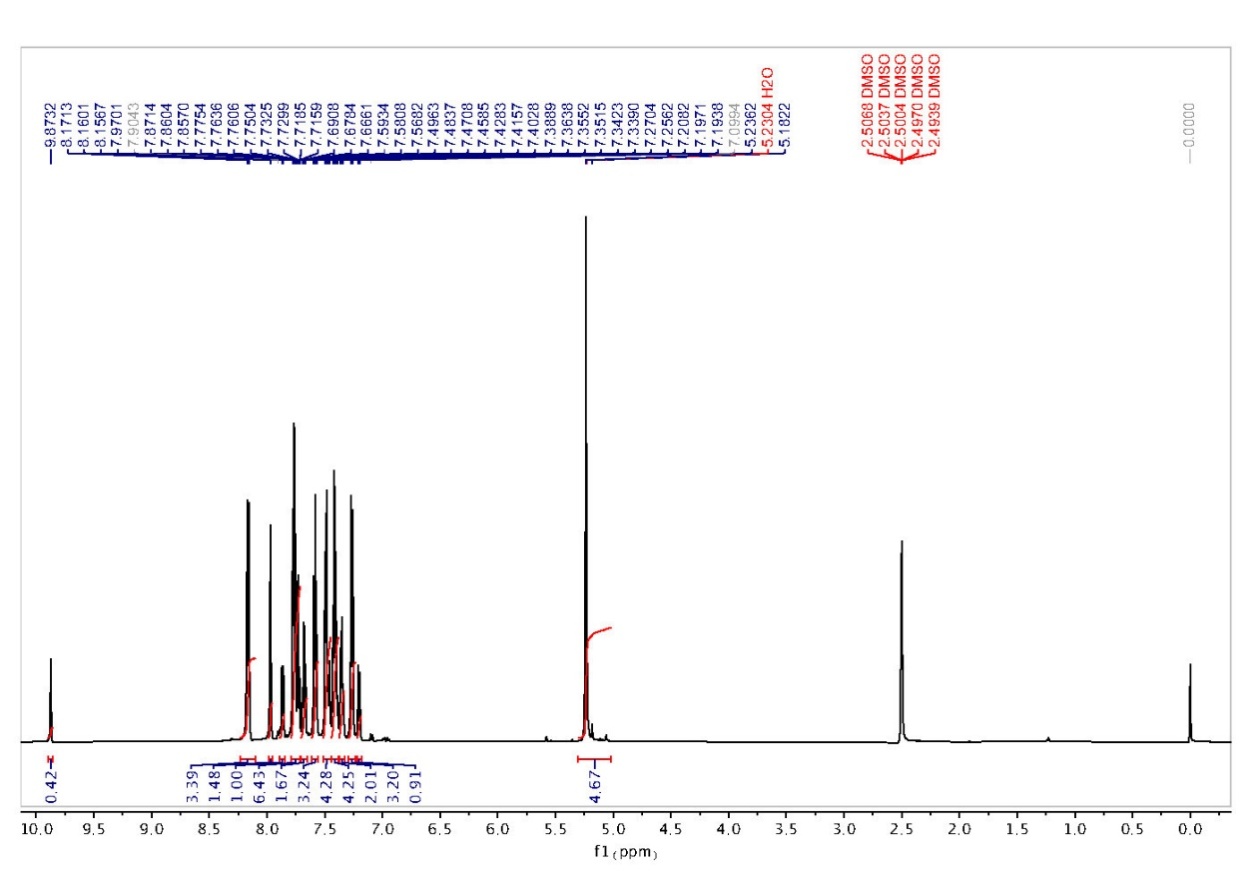


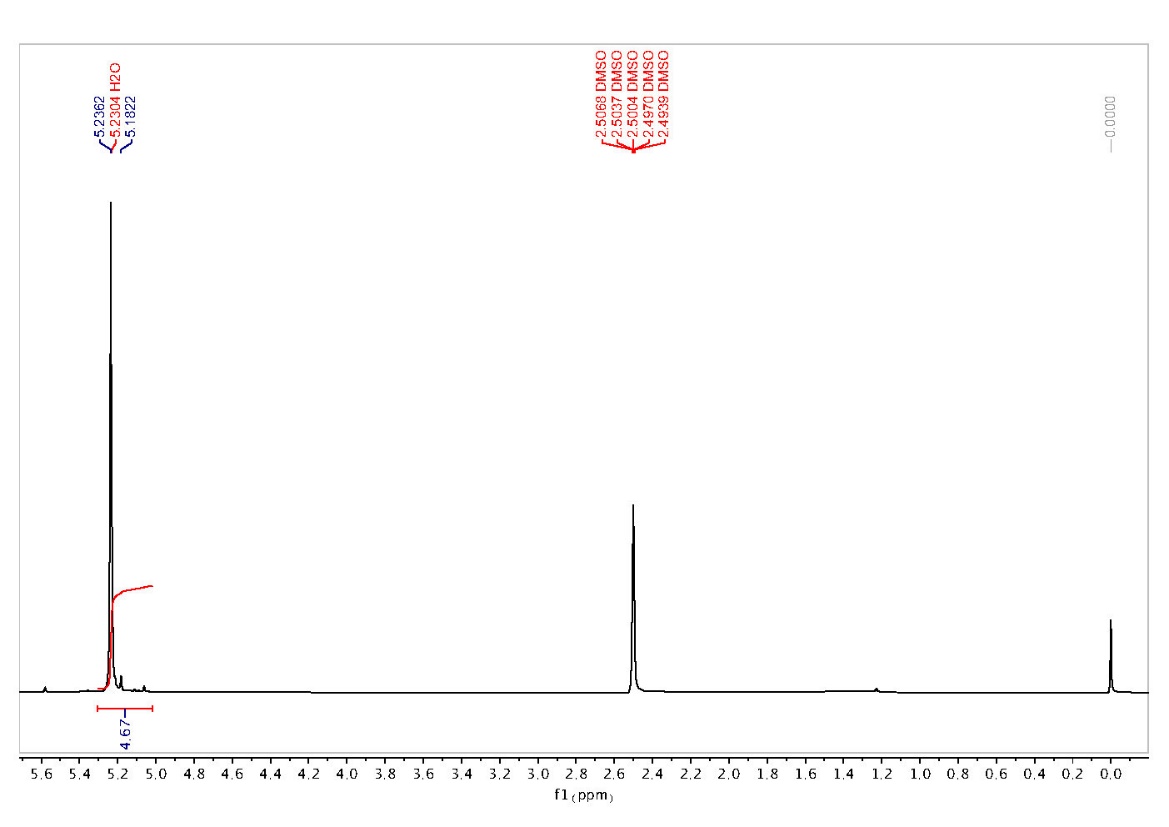


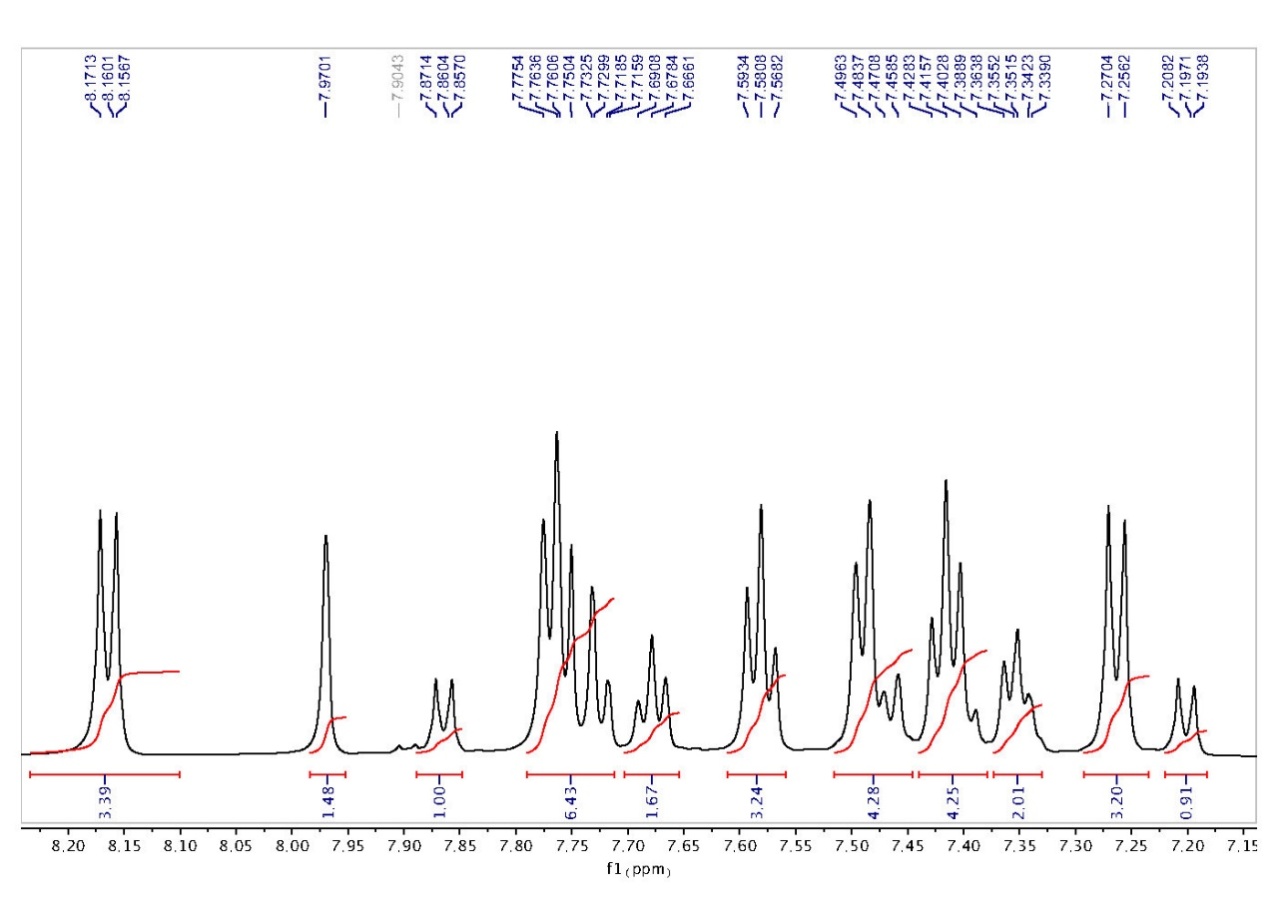


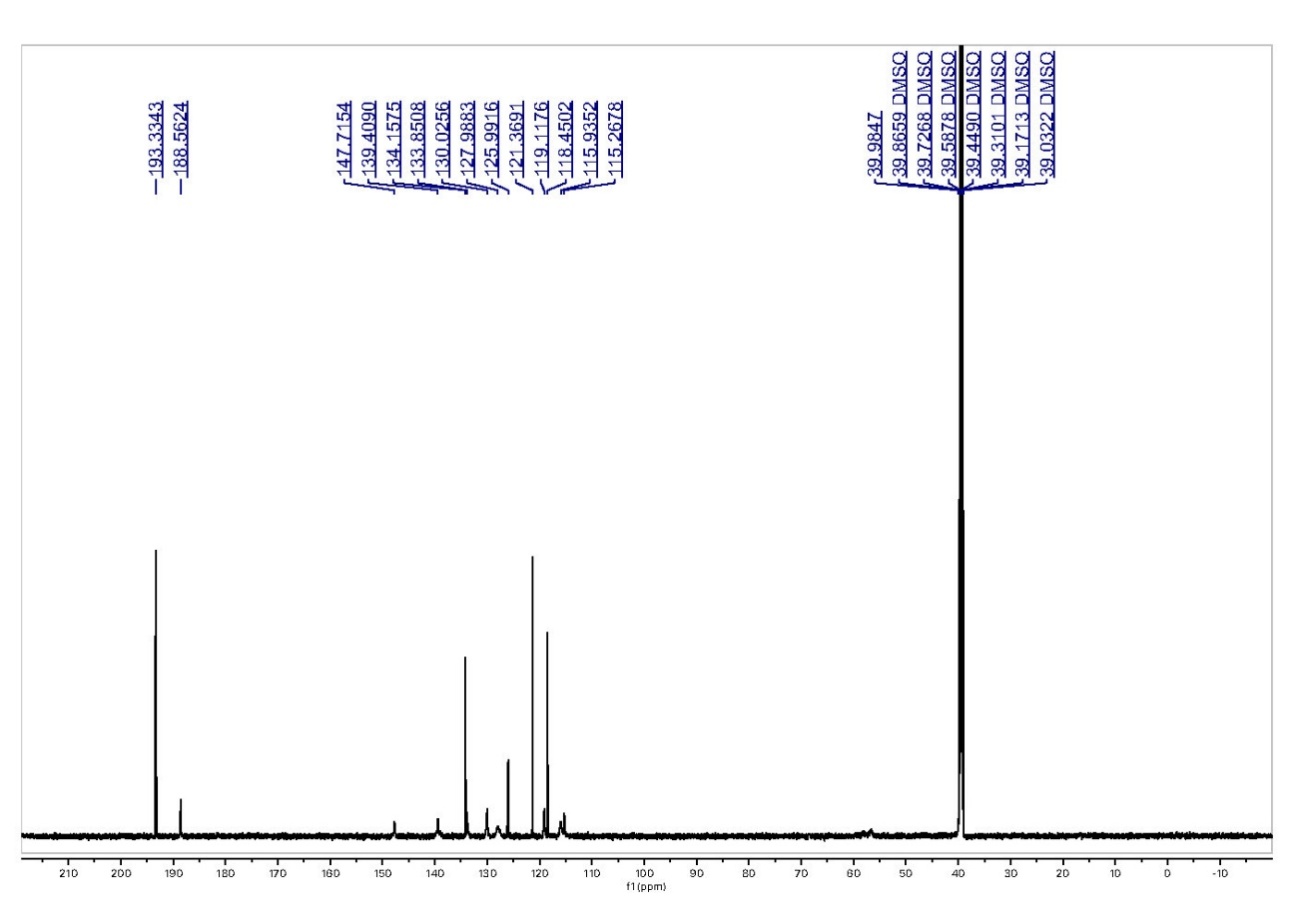


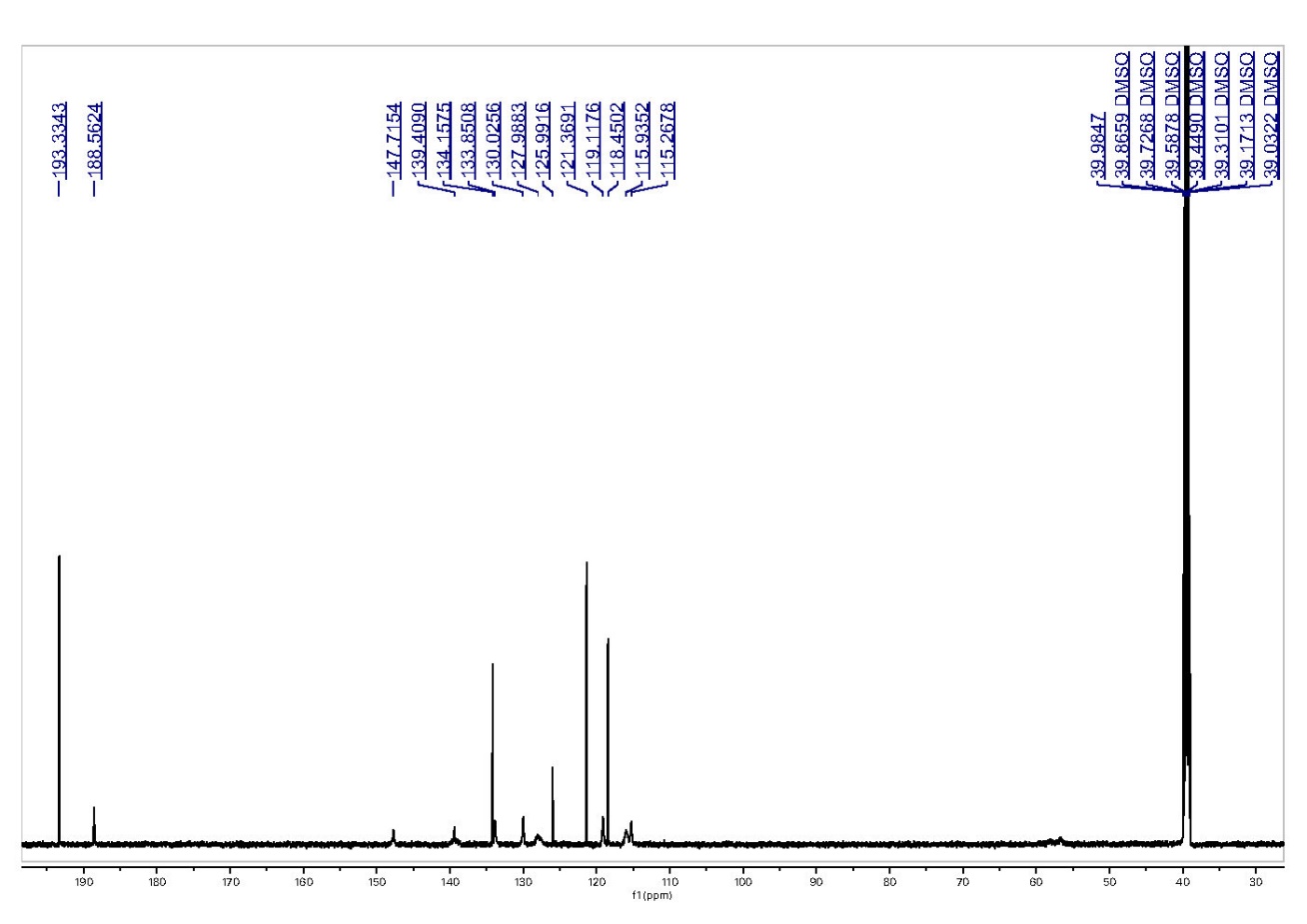


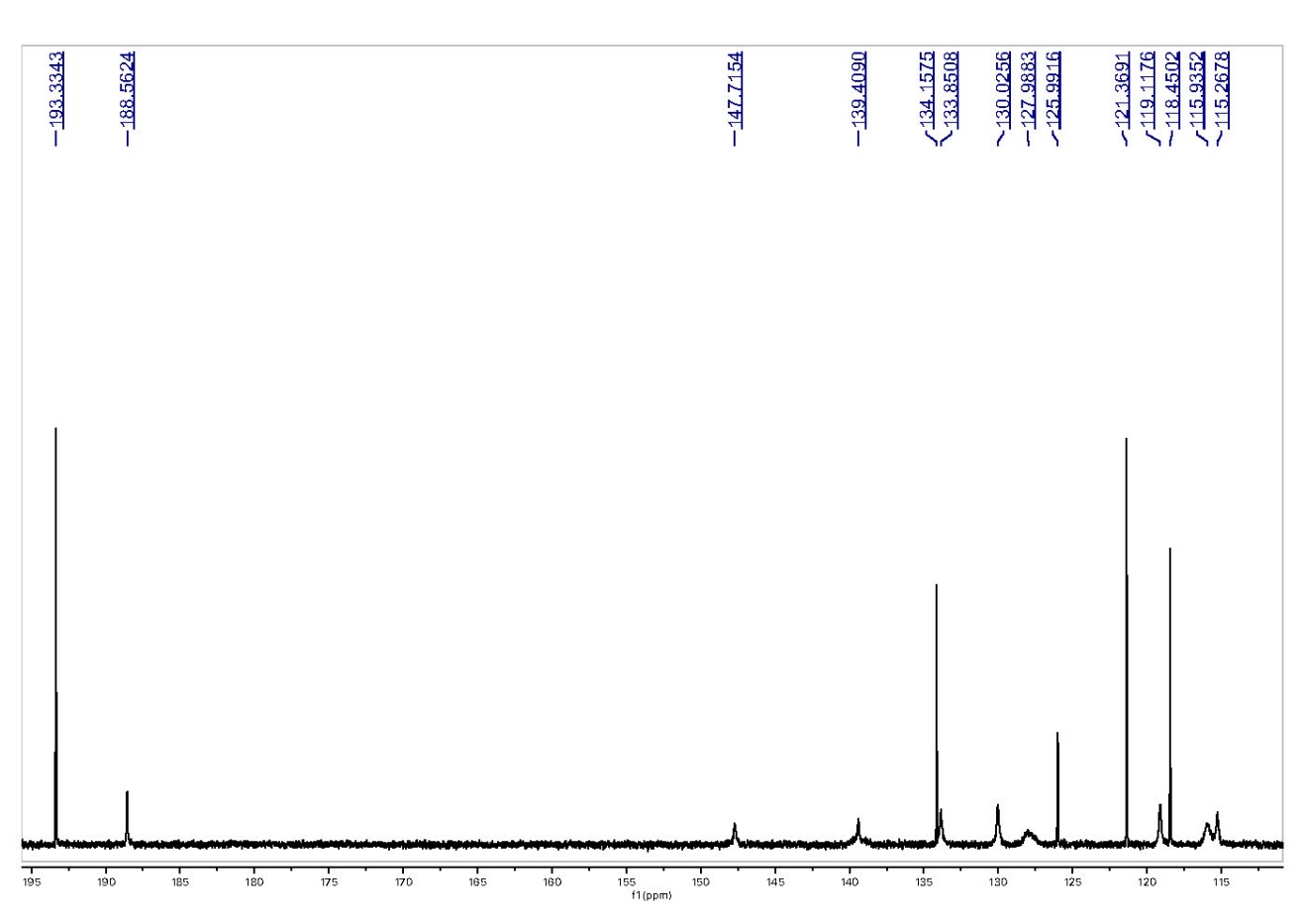

Supplement: Supplementary file 1 [file DataSheet1.docx]
